# Interfacial Hopping Integral as a Predictive Descriptor for Electron Transport: Saturated Alkane Junctions

**DOI:** 10.1021/jacs.5c18728

**Published:** 2026-02-03

**Authors:** Hao Howard Peng, Chih-Hsun Lin, Po-Wei Tung, Chun-Wei Lin, Yen-Chang Chiang, Bon-Shen Wang, Ting-Hsuan Ning, I-Chih Ni, Chih-I Wu, Chun-hsien Chen

**Affiliations:** † Department of Chemistry and Center for Emerging Materials and Advanced Devices, 33561 National Taiwan University, Taipei 10617, Taiwan; ‡ Graduate Institute of Photonics and Optoelectronics, National Taiwan University, Taipei 10617, Taiwan

## Abstract

Electron transport across interfaces governs a broad range of fundamental phenomena. Although orbital overlap is recognized as a key determinant, its experimental quantification remains elusive. Here, we establish the interfacial hopping integral (*t*_eld–mol_), quantifying orbital overlap between contacting atoms, as a predictive descriptor of single-molecule conductance in a benchmark domain of saturated α,ω-functionalized alkane junctions. Using scanning tunneling microscopy and molecular-junction mapping technique, we correlate conductance with molecular tilt (*tilt*_mol_) across π- and σ-type headgroups to extract *t*_eld–mol_. We start with single-atom-thick bismuth and lead adlayers on gold, with dominant *p*-character simpler than gold’s *d*-orbitals. A tight-binding model incorporating Newns–Anderson–Grimley theory yields conductance heatmaps that qualitatively match experiment results and generalize to diverse molecular junctions. Applying this model to the seminal case of alkanedithiols rationalizes literature findings of one to three conductance sets by linking them to *tilt*_mol_ and corresponding *t*_eld–mol_ variations.

## Graphical abstract

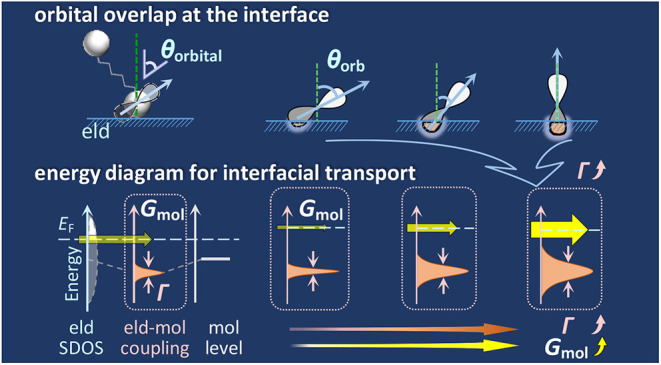

## Introduction

Electron transport across electrode-molecule interfaces underpins a wide spectrum of scientific disciplines. While the hopping integral is well recognized as essential for interfacial electron transport, its measurement remains inaccessible due to the lack of any observable tunable by interfacial parameters. Theoretical models often use an off-diagonal Hamiltonian (Ĥ_eld–mol_, Figure 1a) to describe the electrode-molecule contact. Within this framework, the interfacial hopping integral (*t*_eld–mol_ = ⟨Φ_eld_|Ĥ_eld–mol_|Φ_mol_⟩) quantifies this coupling and governs transport efficiency.^1^ In a nearest-neighbor tight-binding approximation, *t*_eld–mol_ reduces to the overlap of primitive atomic orbitals (AOs) at the contact (Figure 1b). We propose *t*_eld–mol_ as an intuitive descriptor for electron transport and develop an experimental framework to benchmark its predictive power within saturated alkane junctions.

**Figure 1:**
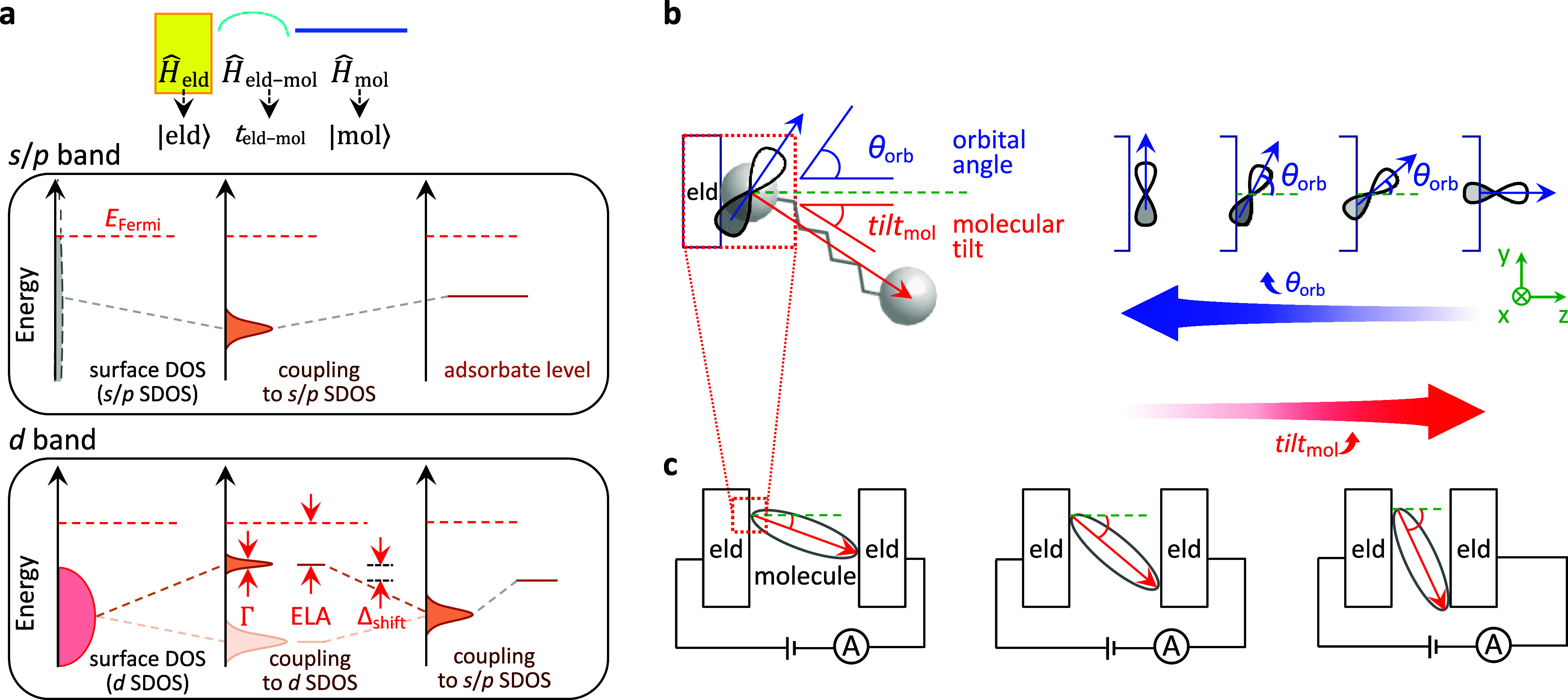
Interfacial hopping integral and its dependence on molecular tilt angles. (a) Schematic of Hamiltonians for electrode–molecule interfaces illustrating discrete adsorbate level coupled with electrodes of (middle panel) *s*/*p* and (lower panel) *d* surface bands. Symbols Δ_shift_, Γ, and ELA denote energy shifts arising from adsorption, level broadening, and energy difference relative to the electrode *E*_Fermi_, respectively. (b) Diagrams illustrating *θ*_orb_, the angle of an atomic *p* orbital with respect to the surface normal. *θ*_orb_ determines the extent of orbital overlap at the interface and is directly related to the molecular tilt angle (*tilt*_mol_) in the laboratory frame. The depicted orientation of the *p* orbital relative to the headgroup-carbon bond is a conceptual illustration based on the calculated FMOs for representative molecular headgroups employed in this study (see Figures 3b or S16). (c) Illustration of molecular *tilt*_mol_ in an EME junction. Reducing the gap spacing increases *tilt*_mol_ angles.

Electric conductance across an electrode-molecule-electrode (EME) junction (Figure 1c) represents an observable of electron transport. A small change in *t*_eld–mol_ might affect the interfacial transport pronouncedly because the junction conductance roughly scales as the fourth power of *t*_eld–mol_,^1^ based on Fermi’s golden rule inherited in the Breit-Wigner type electron transmission^1^ for the Landauer formula.^2^ Apparent parameters enabling the modulation of *t*_eld–mol_ via orbital overlap include the interatomic distance (*d*_nn_) and the angle between the AOs (*θ*_orb_) by a tilt of the molecular chain (*tilt*_mol_). In practice, *d*_nn_ cannot be adjusted with reasonable control, whereas it is experimentally feasible for the latter. Figure 1c illustrates that *tilt*_mol_ (and thus *θ*_orb_, Figure 1b) increases by narrowing the electrode gap spacing (*d*_gap_) which is adjustable using a piezo-driven tip of a scanning probe microscope.^3–10^

Conductance measurements have been performed for EME platforms with monolayer assemblies or single molecules. We choose single-molecule junctions to avoid collective effects present in monolayers, for example, a very limited *tilt*_mol_ range due to steric hindrance in closely packed molecules, extra tunneling paths via neighboring molecules,^5–7^ and interface dipoles that shift the adsorbate’s frontier molecular orbital (*E*_FMO_).^7^ To configure single-molecule junctions, we adapt the molecular-junction mapping (MJM) technique developed by Chang and co-workers^10^ with a tip of scanning tunneling microscope (STM) moving slowly (<1 nm/s) toward or away from the substrate. The slow motion exerts nearly no disturbance to the junction such that the measurement resembles a survey over the EME gap spacings where the bridged molecule can freely adjust suitable *tilt*_mol_ and binding geometries^10^ (Figures 1c and 2a).

**Figure 2:**
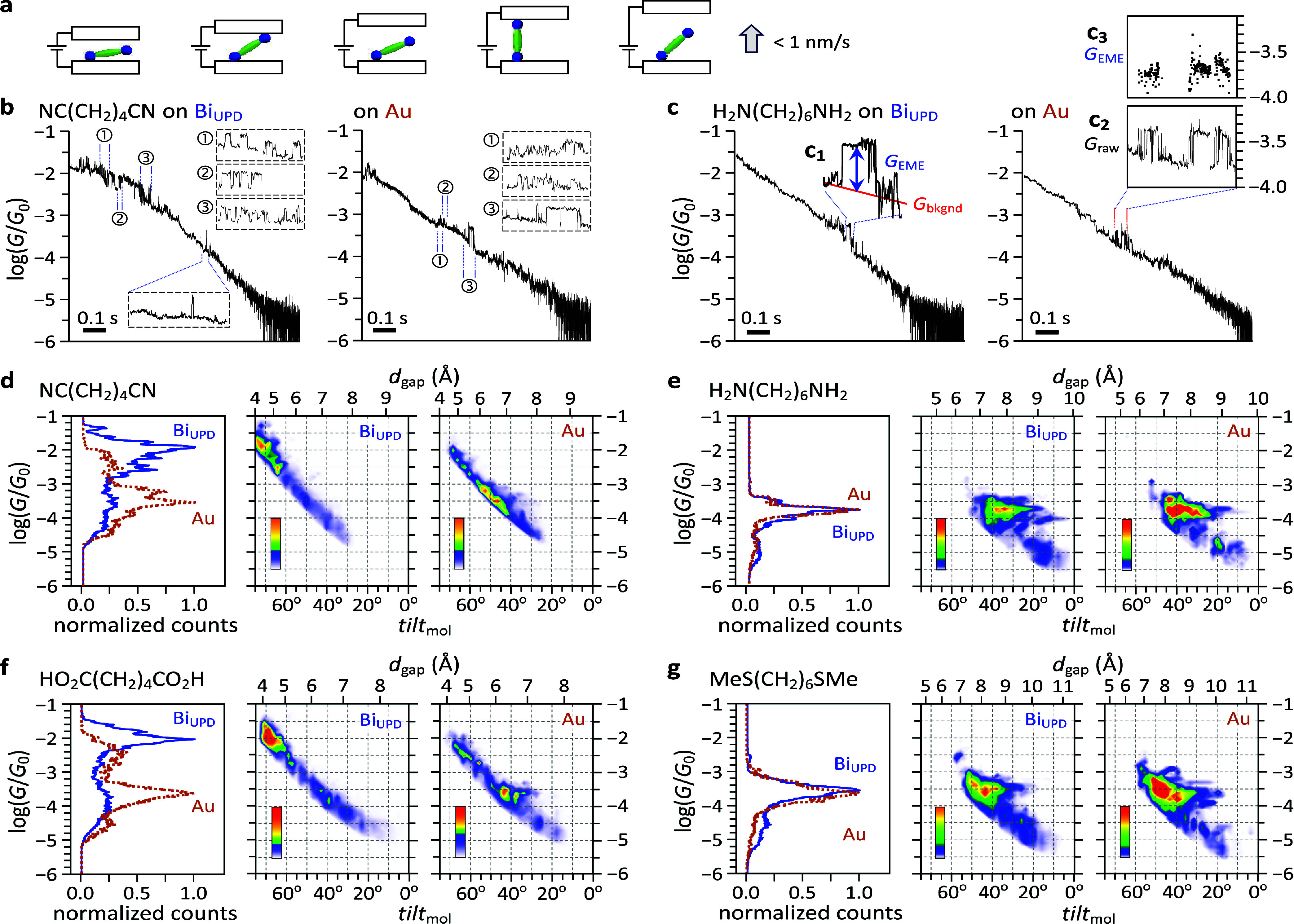
Mapping conductance and molecular tilt via MJM technique. (a) Schematic of the MJM setup. An STM tip moves slowly to modulate the gap spacing between electrodes and allow free formation of EME junctions. (b, c) Representative conductance traces of (b) NC­(CH_2_)_4_CN and (c) H_2_N­(CH_2_)_6_NH_2_ measured on Bi_UPD_ and bare Au electrodes. The magnified sections and insets in panel b emphasize the frequency of conductance jumps and their dwell-time durations, which explain the 2D conductance histograms presented in panel d. Insets (c_1_–c_3_) illustrations of conductance assignment: (c_1_) reported *G*_EME_ value, (c_2_) raw conductance section, (c_3_) background-subtracted section, where *G*_EME_ is extracted from the plateau level following a spontaneous conductance jump above the tunneling background (*G*_bkgnd_); accordingly, the background-subtracted values in c_3_ are lower than the raw data in c_2_. (d–g) Normalized conductance histograms plotted against *tilt*_mol_ and *d*_gap_ for (d) NC­(CH_2_)_4_CN, (e) H_2_N­(CH_2_)_6_NH_2_, (f) HO_2_C­(CH_2_)_4_CO_2_H, and (g) MeS­(CH_2_)_6_SMe. *d*_gap_ is derived from *G*_bkgnd_ and *tilt*_mol_ is calculated from *d*_gap_ and the full-extended molecular length. Other conditions: solvent, trimethylbenzene; tip moving speed, 0.50 nm/s. Upper limits for the color-bar scales (values × 10^3^): Bi_UPD_: (d) 3.0, (e) 0.9, (f) 2.0, (g) 1.8; bare Au: (d) 3.0, (e) 0.5, (f) 1.3, (g) 0.7.

Electrode material offers another dimension to study *t*_eld–mol_, though practical choices are limited to coinage metals, with gold overwhelmingly preferred for its unparalleled malleability in forming EME junctions and for its resistance to oxidation. However, valence states of transition metals feature *d*-orbitals, which are more complicated than *p*-orbitals in terms of shapes and total amounts of magnetic quantum number $m_{l}$ . Additionally, *s/p*-bands are flat compared to *d*-bands. Figure 1a highlights this contrast: on *s*/*p*-band electrodes the adsorbate level is broadened, whereas on *d*-band electrodes it splits into bonding and antibonding states.^11–13^ Hence, we start from electrodes with dominant *p*-character by placing a monoatom-thick Bi or Pb adlayer onto gold electrochemically via underpotential deposition (UPD, abbreviated as Bi_UPD_ ^14^ and Pb_UPD_ ^15^).

To comprehensively assess *t*_eld–mol_, we examine homologous series of α,ω-alkanes with σ- and π-type headgroups. These polymethylene backbones bear *E*_HOMO_ and *E*_LUMO_ located further away from *E*_Fermi_ than *E*_FMO_ of the electrode-headgroup adsorbate state, allowing the use of a single-level model to elucidate their transport behavior. Because the transporting FMO is largely localized on the headgroup (see Figures 3b or S16), the orbital overlap with the electrode can be depicted directly. This interaction can be defined by considering the AOs of the contacting electrode atom and the headgroup atom, thus expressing *t*_eld–mol_ specifically as *t*_eld–head_. Accordingly, the present framework models junctions in which transport is dominated by a single headgroup-localized interfacial state, and where *tilt*_mol_ serves as a geometric descriptor within the MJM analysis.

**Figure 3:**
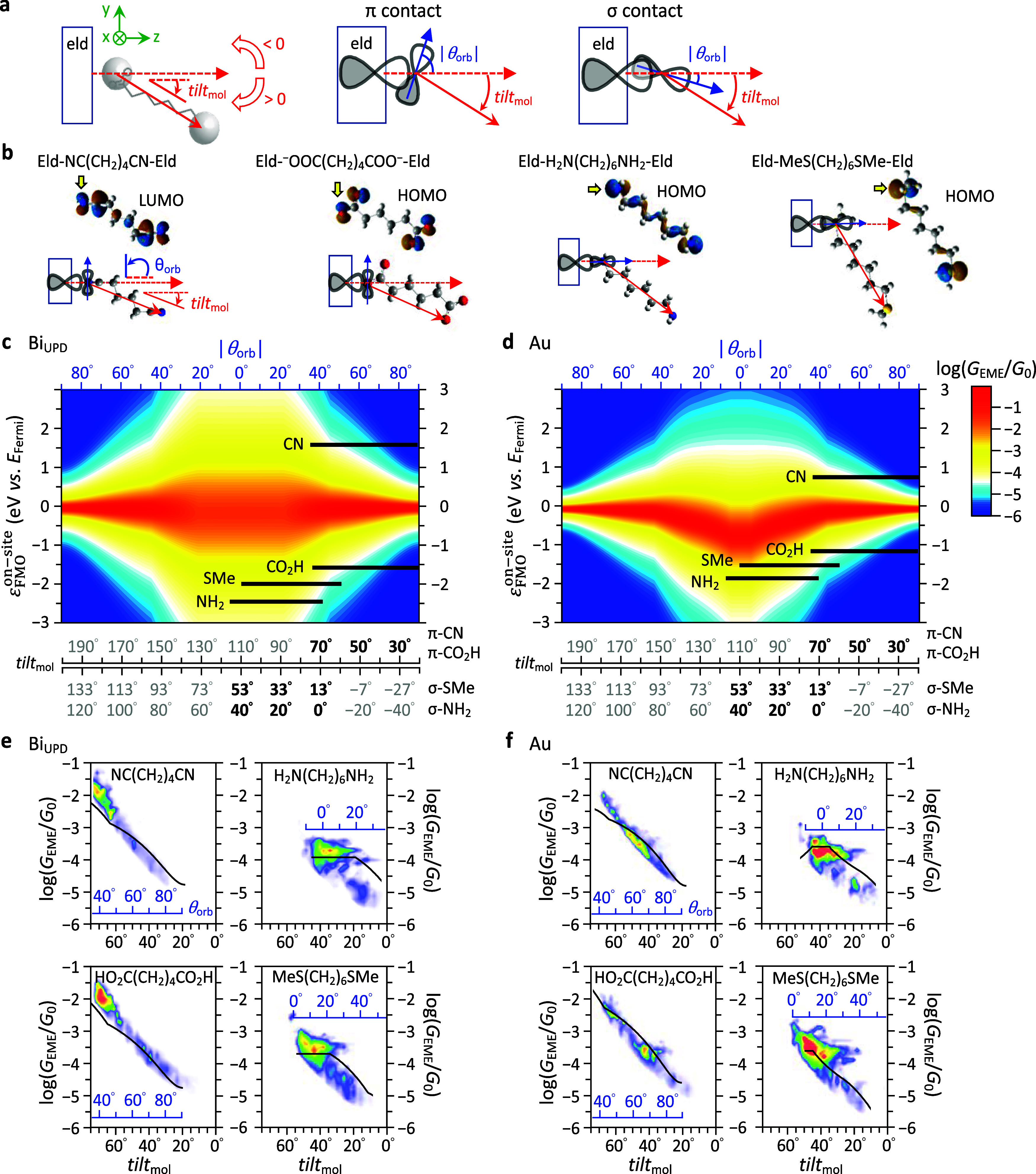
Theoretical conductance heatmaps and the predictivity. (a) Translation from molecular tilt angle *tilt*_mol_ to the degree of electrode-headgroup orbital overlap, where *tilt*_mol_ is defined as the angle between the surface normal and the molecular axis (solid red arrows, defined as the vector from the headgroup end-atom on the surface to the counterpart). For π and σ-type headgroups, the same *tilt*_mol_ results in different *θ*_orb_ and thus different interfacial hopping integrals. (b) FMO of α,ω-alkanes and interfacial-coupling schematic. The FMO for NC­(CH_2_)_4_CN is LUMO^26–29^ and for the others is HOMO. The yellow arrows highlight the FMO densities that contact the electrodes, which are portrayed as *p*-like orbitals in each schematic. Conductance heatmaps for (c) Bi_UPD_ and (d) Au EME junctions. The *y* axis is ε_FMO_^on‑site^ (eV vs *E*_Fermi_), the on-site FMO energy of α,ω-alkanes. The lower *x* axes are *tilt*_mol_ for π and σ headgroups, and the upper *x* axes are *θ*_orb_. The *θ*_orb_ correspondence to *tilt*_mol_ is sketched for each headgroup. (e, f) Overlaid *G*_EME_–*tilt*_mol_ plots (black solid traces in Figure 2d,e) with conductance profiles predicted by the black horizontal lines on the heatmaps (Figure 3c,d). The *tilt*_mol_ range of the conductance profile is limited by steric hindrance. Color scale upper limits for (e, f) are identical to the corresponding electrode–headgroup pairs in panels (d–g) of Figure 2.

Finally, we integrate Newns–Anderson–Grimley model for molecular adsorption^16,17^ with the Slater–Koster^18^ and Harrison^19,20^ formalisms for orbital coupling to construct a tight-binding transporting framework at the atomic orbital level.^21–23^ This model highlights the role of *t*_eld–head_ in electron propagation across EME junctions and produces conductance heatmaps that qualitatively portray junction conductance with the on-site energy of the molecular FMO (ε_FMO_^on‑site^) using *t*_eld–head_ as the key descriptor.

## Results and Discussion

### Conductance-Molecular Tilt Mapping

MJM conductance traces are plotted semilogarithmically versus time (Figure 2b,c). The background current decays exponentially with the EME gap distance (*d*_gap_), described by *i*_bkgnd_ = *G*_bkgnd_ × *V*_bias_ ∝ *V*_bias_ e^–β*d*_gap_^ where β is a tunneling decay constant. Therefore, *i*_bkgnd_ can be used to extract *d*_gap_ (Supporting Information, Section 2-2). In the logarithmical plots (Figure 2b,c), the MJM baselines appear linear-like because *d*_gap_ changed with a constant tip motion (0.50 nm/s). The signature of spontaneous EME formation is the appearance of discrete conductance jumps superimposed on the tunneling background (e.g., Figure 2c_1_). The value of EME conductance (*G*_EME_) is determined by subtracting the tunneling background from the conductance trace plateau (e.g., Figure 2c_2,3_). Data are pooled into a 1D histogram to reveal the most probable *G*_EME_. Plotting *G*_EME_ against another measurable parameter yields a 2D histogram revealing correlations hidden in 1D analysis. For example, 1D histograms of NC­(CH_2_)_4_CN (Figure 2d) show that the most probable peak on Bi_UPD_ is two-order-of-magnitude more conductive than that on bare Au electrodes. Incorporating *tilt*_mol_ confers 2D histograms and reveals that the conductance peak on Bi_UPD_ in the 1D histogram occurs mainly at *tilt*_mol_ ∼ 70°, whereas on bare Au, it occurs at a smaller *tilt*_mol_ near 45°, with a shoulder at ∼53°. These findings match jump frequency and dwelling time distribution (e.g., Figure 2b) although jumps are stochastic and intermittent throughout an MJM trace.

Panels d,f and e,g of Figure 2 exemplify π- and σ-type headgroups, respectively. NC­(CH_2_)_4_CN and HO_2_C­(CH_2_)_4_CO_2_H (π-type headgroups, Figure 2d,f) exhibit similar trends that their most probable *G*_EME_ takes place at larger *tilt*_mol_ on Bi_UPD_ and is significantly more conductive on Bi_UPD_ than on Au. Conversely, σ-type headgroups (−NH_2_ and −SMe, Figure 2e,g) yield comparable conductance on Bi_UPD_ and Au electrodes. Their *G*_EME_ values appear insensitive to increased *tilt*_mol_, in contrast to the increasing patterns observed for π-type headgroups. Near-vertical orientation (*tilt*_mol_ 10–20° for H_2_N­(CH_2_)_6_NH_2_, 20–30° for MeS­(CH_2_)_6_SMe) yields *G*_EME_ ∼one-order-of-magnitude smaller than dominant peaks in 1D histograms, corresponding to the low conductance set assigned in literature reports.^24^

In Figure 2d–g, *G*_EME_ increases with *tilt*_mol_ at small angles. At larger *tilt*_mol_, the increase persists in Figure 2d,f but levels off in Figure 2e,g. Intriguingly, for H_2_N­(CH_2_)_8_NH_2_ (Figure S7), a distinct downturn appears: for *tilt*_mol_ > 30°, *G*_EME_ decreases by nearly half an order of magnitude. This decline likely continues at larger *tilt*_mol_ (i.e., narrower *d*_gap_), but is experimentally masked by the rising background tunneling current (*i*_bkgnd_), which obscures the smaller molecule-mediated transport current, rendering *G*_EME_ inaccessible at high *tilt*_mol_. These trends presented in this section are also observed on Pb_UPD_ (see Figure S14).

### Tight-Binding Transport Model with Headgroup-Orbital Orientation

Under the tight-binding framework,^21–23^ only orbitals overlapping at the electrode-headgroup contact are considered for interfacial propagation. To rationalize the *tilt*_mol_-dependence of *G*_EME_ (e.g., Figure 2d–g) and contrasting behaviors of π- and σ-type headgroups, we apply Harrison’s matrix element *V* (in eV)^19,20^ following Slater and Koster^18^ to describe *t*_eld–head_

1 $$t_{\mathrm{eld}‐\mathrm{head}}=V\cdot \cos\theta_{\mathrm{orb}}$$

where *V* includes an orbital structure factor^19^ relevant to the extent of orbital overlap between atoms at a given *d*_nn_. *V* corresponds to the maximal *t*_eld–mol_ value with the largest orbital overlap at *θ*_orb_ = 0°.

We derive a *t*_eld–head_-integrated quantum transport model from Landauer formalism^2^ (with Meir-Wingreen’s method^25^) and the Newns–Anderson 1D atom-chain-electrode model.^16^ Equation 2 shows the general expression for *G*_EME_ under small *V*_bias_, i.e., *E* ≈ *E*_Fermi_.

2 $$G_{\mathrm{EME}}=G_{0}\frac{\Gamma_{\mathrm{eld}‐\mathrm{head}}^{2}{|}_{E=E_{\mathrm{Fermi}}}}{{\left[{\left(E_{\mathrm{Fermi}}-\varepsilon_{\mathrm{FMO}}^{\mathrm{on}‐\mathrm{site}}-\Delta_{\mathrm{shift}}\right)}^{2}+\frac{\Gamma_{\mathrm{eld}‐\mathrm{head}}^{2}{|}_{E=E_{\mathrm{Fermi}}}}{4}\right]}^{2}}\cdot Ae^{-\beta L}$$

where ε_FMO_^on‑site^ denotes the on-site energy of the molecular FMO, Δ_shift_ is the energy shift of the adsorbed FMO (Figure 1a), *E*_Fermi_ – ε_FMO_^on‑site^ – Δ_shift_ defines the adsorbate energy-level alignment (ELA, Figure 1a), the prefactor *A* accounts for intramolecular coupling (for the explicit form, see eq S2), *L* is the molecular length, and Γ_eld‑head_^2^|_*E*=*E*_Fermi__ describes the effect of electrode-headgroup coupling strength at *E*_Fermi_. Γ_eld–head_ correlates with the extents of orbital overlap (i.e., the hopping integral *t*_eld–head_), and is formulated by eq 3.

3 $$\Gamma_{\mathrm{eld}‐\mathrm{head}}=t_{\mathrm{eld}‐\mathrm{head}}^{2}\cdot \frac{\sqrt{1-{\left(\frac{E-\varepsilon_{\mathrm{eld}}}{2t_{\mathrm{eld}}}\right)}^{2}}}{t_{\mathrm{eld}}},\;\mathrm{for}\;-2t_{\mathrm{eld}}<E-\varepsilon_{\mathrm{eld}}<2t_{\mathrm{eld}}$$

ε_eld_ and *t*_eld_ denote the electrode’s on-site energy (surface band center) and the hopping integral between neighboring sites of the electrode, respectively. The *d*-band contribution (i.e., Γ_eld–head_) vanishes when *E* – ε_eld_ lies outside ±2*t*_eld_. Substituting eq 1 into eq 3, and then into eq 2 shows that *G*_EME_ depends on *θ*_orb_, and thus on *tilt*_mol_.

The *t*_eld–head_-dependent model provides a quantitatively good fit to each MJM-acquired α,ω-alkane *G*_EME_ data on bare Au, Bi_UPD_, and Pb_UPD_, supporting *t*_eld–head_ as a quantitative descriptor of single-molecule junction conductance (Figure S12). Equation 1 adopts a nearest-neighbor approximation in which only the direct electrode–headgroup overlap is treated. In practice, additional through-space tunneling pathways (e.g., to nearby nonadjacent atoms at large *tilt*_mol_) and local surface roughness are not included and may lead to deviations from a pure cosine dependence. Accordingly, the *t*_eld–head_ values extracted from fitting should be hereafter interpreted as effective hopping integrals that subsume these unmodeled contributions. Details of the procedures and fitting results are provided in Supporting Information Section 3 and Table S5.

### Conductance Heatmaps for Interfacial Electron Transport

Figure 3 presents simulated conductance heatmaps, generated from eqs 1–3 in which the required parameters were obtained by fitting MJM data for NC­(CH_2_)_4_CN (π-type) and H_2_N­(CH_2_)_6_NH_2_ (σ-type); the heatmaps use the average of these two sets of fitted parameters (see footnote d of Table S5). The *z*-axis value of Figure 3c,d at each coordinate pair of ε_FMO_^on‑site^ and *tilt*_mol_ represents zero-biased *G*_EME_|_*E*=*E*_Fermi__. Figure 3a depicts a pair of π- and σ-type headgroups with identical *tilt*_mol_, yet their corresponding *θ*_orb_ orientations differ by ∼90° (the angle between the two blue arrows at the central and right panels). Figure 3a also illustrates the correlation between the laboratory-frame *tilt*_mol_ and the molecular-frame *θ*_orb_. Steric interactions with the surface constrain the accessible *tilt*_mol_ range, mainly due to the proximal terminal groups such as the hydrogen atoms in −NH_2_ or the methyl group in −SMe. Figure 3b presents the FMO of α,ω-alkanes and corresponding schematic of interfacial coupling. The FMO responsible for electron transport is LUMO for NC­(CH_2_)_4_CN^26–29^ and HOMO for the others. The FMO densities that contact the electrodes (indicated by yellow arrows) are portrayed as *p* atomic orbitals in each schematic.

Figure 3c, modeled on Bi_UPD_, exhibits *G*_EME_ contours symmetric about the *x*-axis. The red and yellow regions below ε_FMO_^on‑site^ = 0 are marginally outweigh those above, reflecting the slightly negative-positioned Bi *p*-band center. This hardly noticeable asymmetry arises because, for adsorbates with ε_FMO_^on‑site^ in resonance with the Fermi level (i.e., at ε_FMO_^on‑site^ = 0 eV), coupling to the *p* band shifts the FMOs away from *E*_Fermi_, increases their energy offset, and attenuates ELA, thus lowering *G*_EME_ slightly. As *θ*_orb_ decreases from 90°, the red zone disperses due to broadened Γ_eld–head_, indicative of enhanced interatomic orbital overlap and strengthened *t*_eld–head_. Below *θ*_orb_ ∼ 30°, this broadening diminishes because inductive effects (i.e., orbital reorientation)^30–32^ energetically align the interfacial *p*-orbitals and maintain optimal overlap.^12,33^ Hence, further decreasing *θ*_orb_ produces negligible additional broadening of the red zone, unlike the trend on Au (Figure 3d, vide infra). According to the fitting results, this inductive reorientation also shortens *d*_nn_, strengthening coupling to the Bi_UPD_ 6*p*_*z*_-band. Stronger coupling stabilizes the HOMO and destabilizes the LUMO,^34^ increasing |*E*_Fermi_ – *E*_FMO_| (i.e., a poorer ELA) in both HOMO- and LUMO-dominant transport regimes, ultimately reducing *G*_EME_.

Horizontal slices at specific ε_FMO_^on‑site^ values are plotted as *G*_EME_–*tilt*_mol_ traces in Figure 3e,f, overlaid with experimental MJM data. These traces, scaled by *G*_EME_ ∝ e^–βL^ (eq 2) to account for molecular-length differences (i.e., the differences in methylene units), agree well with experimental results. For π-type NC­(CH_2_)_4_CN or HO_2_C­(CH_2_)_4_CO_2_H in Figure 3c, *G*_EME_ increases with larger *tilt*_mol_, attributed to enhanced nitrogen-6*p*_*z*_(Bi_UPD_) or monodentate oxygen-6*p*_*z*_(Bi_UPD_) orbital interactions (see Figures 3b or S16). For σ-type H_2_N­(CH_2_)_6_NH_2_ and MeS­(CH_2_)_6_SMe in Figure 3d, *G*_EME_ rises rapidly as *tilt*_mol_ moves from 0 to ∼ 30° (specifically, *θ*_orb_ decreases from ∼40 to ∼20° for −NH_2_ and from ∼50 to ∼20° for −SMe) and then levels off, reflecting improved orbital alignment and the inductive effect. Remarkably, while this heatmap is prepared by parameters of −NH_2_/–CN headgroups, the cross sections capture conductance trends for −COOH and −SMe. The strong agreement between heatmap-derived traces and experimental data (Figure 3c) highlights the generality and predictive capability of the model across diverse headgroups.

Figure 3d, modeled on bare Au, demonstrates how *d*-bands perturb the *s*/*p*-band-based heatmap of Figure 3c. Compared to Figure 3c, contours in Figure 3d shift downward, and the red zone becomes V-shaped. These features stem from Au’s low-lying, narrow, and fully filled *d*-bands. Au-adsorbate interactions splits the adsorbate state into bonding and antibonding states (Figure 1a), with the antibonding states often found below *E*_Fermi_ and occupied.^11^ Occupation of this antibonding state counteracts bonding stabilization, leading to a net destabilization termed metal-adsorbate repulsion.^11,35,36^ An increased orbital overlap toward small *θ*_orb_ reinforces this repulsive effect, impedes the inductive orbital reorientation, and reduces the tendency for EME junction formation, although the improved ELA does raise *G*_EME_.

For a quantitative perspective, Nørskov et al. describe the chemisorption energy (Δ*E*_d–hyb_) as^12^

4 $${\Delta E}_{d‐\mathrm{hyb}}=-2\left(1-f_{d}\right)\frac{V^{2}}{\left|\varepsilon_{d}-\varepsilon_{\mathrm{ab}}\right|}+2\left(1+f_{d}\right)\alpha V^{2}$$

where the first term represents attractive orbital mixing and the second term represents Pauli repulsion. *V* denotes the metal-adsorbate coupling matrix element (equivalent to *t*_eld–head_), ε_d_ and ε_ab_ are the energies of the metal *d*-band center and the antibonding state (equivalent to ε_FMO_^on‑site^ + Δ_shift_), respectively. α is a positive constant, accounting for the energy penalty of overlapping electron orbitals constrained by the Pauli exclusion principle (namely, orbital orthogonalization), and *f*_d_ is the *d*-band filling fraction.^12^

For metals like Au with fully occupied *d*-bands (presumably *f*_d_ = 100%), the attractive term is effectively nullified. However, Abild–Pedersen et al. found that a small fraction of antibonding states remains unfilled.^37^ Hence, Au-adsorbate interactions are dominated by Pauli repulsion, disfavoring EME junction formation despite modest ELA gains. This repulsion suppresses the inductive effect, causing Γ_eld–head_ to broaden gradually with increasing *tilt*_mol_. Consequently, Au electrodes reach Γ_eld–head_ saturation more slowly, extending the inductive response over a wider *tilt*_mol_ range and yielding the V-shaped contour (Figure 3d). Conversely, Bi_UPD_ (*f*_d_ = 0), free of antibonding penalties, enables rapid Γ_eld–head_ saturation, producing plateau-like conductance behavior and a stronger inductive response. Accordingly, uHC is suppressed on Au (near *d* ^10^) but prominent on Bi_UPD_ and Pb_UPD_, consistent with stronger filled-*d*-band repulsion on Au under high-*tilt*_mol_ configurations that yield larger *t*_eld–head_.

For −CN/–COOH headgroups (π-type), *G*_EME_–*tilt*_mol_ trends on Au are similar to those on Bi_UPD_. However, the *G*_EME_–*tilt*_mol_ plots (Figures 2d,f and 3e,f) shows that high-tilt configurations occur less frequently on Au (also consistent with the EME events shown in Figure 2b), likely due to the above-mentioned repulsion at *tilt*_mol_ > 55°.

For −NH_2_/–SMe (σ-type headgroup), *G*_EME_ rises as *tilt*_mol_ increases from ∼5 to ∼35°, reflecting inductive orbital alignment and *d*_nn_ adjustment. Greater than ∼35°, conductance does not increase and even declines for H_2_N­(CH_2_)_8_NH_2_ (Figure S7).

These conductance heatmaps provide qualitative predictions for each headgroup–electrode pair. Notably, the Bi_UPD_ and Pb_UPD_ benchmark heatmaps are closely similar (Figures S13 and S14), consistent with both being *p*-character electrodes. Their *p*-band centers lies slightly below (Bi_UPD_) and above (Pb_UPD_) *E*_Fermi_, which subtly shifts the high-conductance region (red zone) toward the lower and upper halves of the heatmap. However, for the headgroups studied here, the fitted ε_FMO_^on‑site^ values are away from *E*_Fermi_; therefore, this modest landscape shift does not lead to discernible conductance difference.

Using our benchmark systems, four types of headgroups measured on bare Au, Bi_UPD_, and Pb_UPD_, as references, untested pairs can be anticipated from three inputs: (i) the electrode surface DOS (e.g., Figure S10), which shapes the heatmap landscape; (ii) the headgroup’s orbital symmetry (π or σ) and steric constraints, which define the accessible tilt range (bottom *x*-axis); and (iii) the on-site frontier level, ε_FMO_^on‑site^ (*y*-axis). In our implementation, ε_FMO_^on‑site^ values are determined by fits to MJM data (Table S5). For untested systems, qualitative placement follows the dominant frontier orbital: LUMO-dominant (n-type) responses map to the upper half of the heatmap, whereas HOMO-dominant (p-type) responses map to the lower half; within each half, the conductance profiles are closely similar. The Newns–Anderson DOS is represented by a semielliptical form for analytical tractability, differences from the DFT-projected DOS propagate primarily through Γ and the real-part level shift that sets ELA, thereby influencing the absolute conductance scale rather than the trend of tilt angles. An estimate based on Figure S10 suggests that for Au this corresponds to a few-fold uncertainty in *G*_EME_, while the *G*_EME_–*tilt*_mol_ dependence governed by *t*_eld–head_ remains unchanged.

### *t*_eld–head_ as a Descriptor for Alkanedithiol Junctions

Alkanethiols, renowned for their seminal role in self-assembled monolayers and molecular electronics, have long served as the benchmark for methodological development in single-molecule *G*_EME_ measurements.^38,39^ Despite extensive study, reported alkanedithiol *G*_EME_ values vary widely, ranging from no discernible peak^40,41^ to one,^41–46^ two,^47,48^ or multiple^10,43,49–53^ conductance sets (commonly categorized as high (HC), medium (MC), and low (LC)). This variability is attributed to inherent complexities in junction geometries, binding sites, molecular conformations, measurement schemes, experimental conditions, and data analysis protocols. Quantum transport simulations that typically assign HC to bridge^49,53,54^ or hollow^47,53–55^ sites and LC to atop sites^47,49,53–55^ predict only modest 2–5-fold^54,55^ conductance differences, far short of the ∼18–25-fold spread observed experimentally.^10,49,52,53,56,57^ Wandlowski, Evers, and co-workers proposed that LC has to arise from gauche conformers, which are shorter than the fully extended HC conformation and thus should appear at narrower *d*_gap_.^49^ Because STM-BJ traces progress from short to long *d*_gap_, this interpretation predicts LC should precede MC/HC within a single trace; yet, in the same paper the authors report that MC never follows LC, nor does HC follow MC.^49^ Comparisons are further hampered because computational studies often employ diverse, model-dependent electrode–headgroup geometries tuned to reproduce individual experimental results, complicating cross-study benchmarking.^49,54,55^

By suppressing artifacts (e.g., multimolecule bridging or through-space tunneling), controlling *d*_gap_, and correlating *G*_EME_ with *tilt*_mol_, the MJM method employed herein mitigates junction heterogeneity and enables unbiased identification of multiple conductance states in a single measurement.^10^ The resulting 1D histograms and explicit *G*_EME_–*tilt*_mol_ relations motivate a new perspective on this longstanding issue through interpretation with the *t*_eld–head_-based model.

MJM-acquired 1d-histograms for octanedithiol (Figure 4a_1_) show peak values comparable to literature reports;^10,49,56,57^ accordingly, we label the peaks following the established assignment.^10,42^ Figure 4a_2_,a_3_ show that HC, MC, and LC emerge sequentially as *d*_gap_ increases,^24,49^ reflecting increasing *θ*_orb_ (Figure 4b_1_). The *G*_EME_–*tilt*_mol_ profiles of octanedithiol mirrors those of π-type headgroups and aligns with the alkanethiol HOMO, which has a π-like lobe at sulfur^55,58^ (Figure S16). On bare Au, HC and LC are less populated than MC, while Bi_UPD_ shows more pronounced HC. The suppressed HC on Au likely reflects antibonding orbital repulsion.

**Figure 4:**
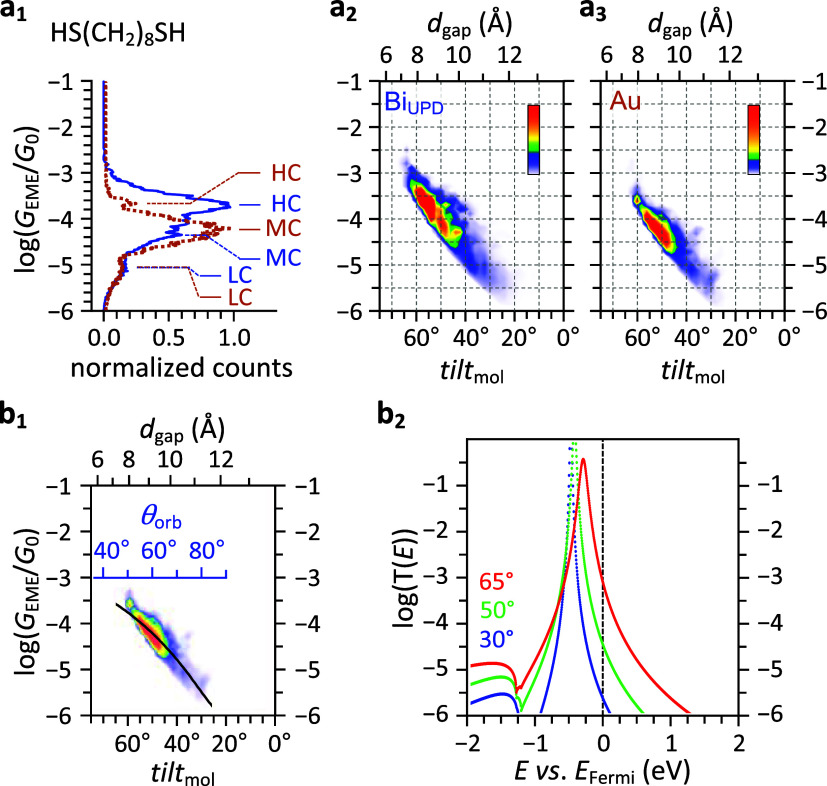
Conductance-tilt mapping and *t*_eld–head_-based model validation for HS­(CH_2_)_8_SH. (a) MJM-acquired conductance histograms of HS­(CH_2_)_8_SH on (a_1_) Bi_UPD_ (blue) and Au (brown) electrodes, and corresponding (a_2,3_) *G*_EME_–*tilt*_mol_ plots showing HC, MC, and LC conductance taking place sequentially with increasing *d*_gap_. (b) *t*_eld–head_-based modeling. (b_1_), Simulated *G*_EME_–*tilt*_mol_ profile at ε_FMO_^on‑site^ = −0.57 eV (vs *E*_Fermi_) of the heatmap in Figure 3d, overlaid with MJM data, reproduces the experimental trend. (b_2_) Associated transmission spectra show that increasing *tilt*_mol_ broadens Γ_eld–head_, improves ELA, and raises *T*(*E*_Fermi_). These results support *t*_eld–head_ as a quantitative descriptor of interfacial coupling in alkanedithiol junctions. Upper limits for the color-bar scales: Bi_UPD_: (a_2_) 3.0 × 10^3^; bare Au: (a_3_, b_1_) 1.5 × 10^3^.

Fitting the MJM data to the *t*_eld–head_-based model for alkanedithiols gives ε_FMO_^on‑site^ = −0.57 eV (Table S5). The corresponding *G*_EME_–*tilt*_mol_ curve from the heatmap reproduces the experimental trend (Figure 4b_1_). Concomitantly, the model’s transmission spectra (Figure 4b_2_) show that increasing *tilt*_mol_ significantly broadens Γ_eld–head_, slightly improves ELA, and raises the transmission at *E*_Fermi_. This *G*_EME_–*tilt*_mol_ correspondence (Figure 4b_1_) supports the applicability of the *t*_eld–head_-based model for describing the effective interfacial coupling of alkanedithiols.

## Conclusion

In summary, we establish the interfacial hopping integral *t*_eld–head_ as a predictive descriptor for electron transport across electrode-molecule interfaces. We developed a simple atomic-orbital framework (inspired by Slater–Koster^18^ and Harrison^19^ models) and validated it using α,ω-alkanes bridging electrodes with *p*-character Bi_UPD_ and Pb_UPD_, and subsequently applied it to Au, the de facto standard in this field, to address its *d*-band complexity. By employing the MJM technique to vary molecular tilt, we directly modulated the interfacial orbital overlap and thereby tuned the junction conductance.

This approach yielded three key findings: (1) unexpectedly high conductance (≈10^–2^ *G*_0_) for NC­(CH_2_)_4_CN and HO_2_C­(CH_2_)_4_CO_2_H on *p*-character electrodes, far exceeding the ∼10^–4^ *G*_0_ typical for similar-length alkanes; (2) distinct conductance profiles between π-type and σ-type headgroups; and (3) a remarkable 2–3-order-of-magnitude conductance span governed by molecular tilt. Guided by these insights, we constructed a conductance heatmap of *G versus* frontier-level energetics and molecular tilt angle, the latter directly tuning *t*_eld–head_. This model reproduces all observed conductance trends across various headgroups and electrode surfaces. It also explains the longstanding puzzle of multiple conductance values in alkanedithiol junctions by linking them to different tilt configurations, and offers a predictive blueprint for designing and understanding electron transport in diverse molecular interfaces. Finally, the conductance heatmaps presented here can serve as MO-wise building blocks (i.e., one map per molecular orbital). In principle, superimposing these maps following established quantum circuit or interference rules could provide a practical route to extend the framework to multiorbital transport as a function of the geometric descriptor.

## Methods

### Fabrication of Bi_UPD_ and Pb_UPD_ Modified Gold Electrodes

All chemicals were of analytical grade and used as received. Bare gold substrates were fabricated by thermally evaporating a 100 nm-thick gold film onto piranha-cleaned glass slides, with a 5 nm-thick chromium adhesion layer in between. *Piranha solution,* 1:3 (v/v) mixture of 30% H_2_O_2_ and 98% H_2_SO_4_, *reacts exothermically and violently with organics. It should be handled with extreme caution*. Prior to experiments, gold substrates were cleaned by UV/ozone treatment (Bioforce, Salt Lake City, UT, USA). Remaining organic contaminants were removed by flame desorption using a butane torch. STM tips were prepared by mechanically cutting gold wires (99.95%, 0.25 mm diameter, Leesan, Tainan, Taiwan) with diagonal pliers. Solutions of 0.1 M HClO_4_ containing 1 mM Bi^3+^ or Pb^2+^ (from Bi_2_SO_3_ or Pb­(ClO_4_)_2_, Sigma-Aldrich) were employed to electrodeposit a single-atom-thick adlayer of bismuth^14^ or lead^17^ onto the gold STM tips and substrates. An Ag/AgCl reference electrode and a platinum-foil counter electrode were used. Gold substrates were potentiostatted at ∼30 mV positive from the Nernst potential of Bi^3+/0^ or Pb^2+/0^ for ≥10 s to deposit a monolayer of Bi or Pb, and withdrawn from solution while maintaining the applied potential.

### MJM Experimental Scheme and Data Analysis

All MJM measurements^10^ were conducted in trimethylbenzene (TMB). A highly diluted surface coverage of α,ω-alkane was used to facilitate the formation of single-molecule EME junctions.^9^ Hence, electrodes were immersed for 30 s in a TMB solution containing ≤10 μM X­(CH_2_)*_n_* X (*n*: 4, 6, 8; X: −CO_2_H (Showa), −NH_2_ (TCI), −SMe (Merck), −CN (Sigma-Aldrich), −SH (Alfa)) and then gently dried under a stream of nitrogen. Subsequently, the electrodes were placed in neat TMB for MJM experiments in which the STM tip approached the substrate at 1–5 nm/s and was retracted at 0.5–0.8 nm/s once the background tunneling reached a preset threshold of 0.10 *G*_0_. For the data analysis, 1000 *i*_EME_(*t*) traces was collected during retraction for each electrode-molecule pair. Because MJM operates in a deliberately low-coverage regime, modest run-to-run variations in adsorption and rinsing can affect junction-formation probabilities and thus the relative peak populations, even for repeated measurements on the same bare Au, Bi_UPD_ or Pb_UPD_ electrodes. Custom LabVIEW programs (National Instruments) were employed to drive STM tip motion, acquire *i*_EME_(*t*), and fit the data using our *t*_eld‑head_-based model.

Molecular jump events were identified using the derivative of conductance-time traces, d log­(*G*/*G*_0_)/d*t* and were screened based on S/N ratio thresholds. Junction conductance values were obtained by subtracting the tunneling background, binning the data into histograms, and applying Gaussian fitting. The 2D conductance heatmaps were obtained by mapping junction conductance as a function of adsorbate on-site energy and molecular tilt angle, calculated with cos^–1^(*d*_gap_/*L*_mol_), where *L*_mol_ is defined as the distance between the two terminal anchoring atoms of the fully extended α,ω-alkane, and *d*_gap_ is the electrode gap distance calibrated via the piezo-driven tip motion against background tunneling with the target molecule present in the solution (see Table S3). With this definition, *tilt*_mol_ should be regarded as an *effective* geometric descriptor rather than a literal molecular tilt.

### Quantum Mechanical Calculations

Density functional theory (DFT) calculations were used to analyze the spatial characteristics of the FMOs of α,ω-alkanes and their role in electrode-molecule coupling. The FMOs were computed in the gas phase to reveal headgroup-localized orbital distributions, which form the basis of the *t*_eld–head_-based model. To obtain the electrode surface density-of-states for fabricating the heatmaps in Figure 3c,d, DFT calculations were performed based on the setups in Supporting Information Section 4-2, and the calculated results are provided in Figure S10.

## Supplementary Material



## Notes

Preparation and characterizations of Bi_UPD_ and Pb_UPD_ bimetallic electrodes, processing and analysis of MJM conductance traces, theoretical modeling and conductance heatmaps, DFT and NEGF-DFT calculations, note on model applicability and additional analyses, and glossary for term definitions (PDF)
