# Supplementary material for: Interfacial Hopping Integral as a Predictive Descriptor for Electron Transport: Saturated Alkane Junctions
Source: J Am Chem Soc. 2026 Feb 3;148(6):6289–98. doi: 10.1021/jacs.5c18728 (PMC12921866; doi:10.1021/jacs.5c18728)
Supplement: Supplementary file 1 [file ja5c18728_si_001.pdf]

Interfacial Hopping Integral as a Predictive Descriptor for Electron Transport: Saturated Alkane Junctions

Hao Howard Peng,[†,§] Chih-Hsun Lin,[†,§] Po-Wei Tung,[†] Chun-Wei Lin,[†] Yen-Chang Chiang,[†] Bon-Shen Wang,[†] Ting-Hsuan Ning,[†] I-Chih Ni,[‡] Chih-I Wu,[‡] and Chun-hsien Chen[*†]

[†] Department of Chemistry and Center for Emerging Materials and Advanced Devices, National Taiwan University, Taipei 10617 (Taiwan)

[‡] Graduate Institute of Photonics and Optoelectronics, National Taiwan University, Taipei 10617 (Taiwan)

1. Preparation and Characterizations of Bi_{UPD} and Pb_{UPD} Bimetallic Electrodes. 2
  1-1. Preparation of Bi_{UPD} and Pb_{UPD} 2
  1-2. Characterizations of Bi_{UPD} and Pb_{UPD} adlayers 2

2. Processing and Analysis of MJM Conductance Traces 5
  2-1. Validation of data processing and results of MJM measurements 5
  2-2. Tunneling background, interelectrode spacing, and G_{EME}–$tilt_{mol}$ heatmaps 9
  2-3. G_{EME}–$tilt_{mol}$ of $H_2N(CH_2)_8NH_2$ with clear signatures predicted by the self-derived model 10

3. Theoretical Modeling and Conductance Heatmaps 13
  3-1. Conductance formula for EME junctions 13
  3-2. Hopping integral $t_{eld\text{-}head}$ derived from Harrison′s and Slater–Koster′s method 16
    3-2-1. Hopping integral for adsorption on *p*- and *d*-block metal electrodes: Harrison′s method. 16
    3-2-2. Angular dependence of hopping integral: Slater–Koster′s method. 18
    3-2-3. Adsorption-induced stick-slip-like θ_{orb} realignment and d_{nn} adjustment. 19
  3-3. Conductance heatmaps: preparation and predictive modeling 20
    3-3-1. Preparation of conductance heatmaps 20
    3-3-2. Prediction capability of the conductance heatmaps for electron transport. 22

4. DFT and NEGF-DFT Calculations 26
  4-1. Gas-phase FMOs and $t_{eld\text{-}head}$-based interfacial transport picture 26
  4-2. EME junction setup and transmission calculations 27

5. Notes on Model Applicability and Additional Analyses 28
  5-1. Layer-resolved PDOS assessment of Au(*d*)–Adlayer(*p*) orbital mixing Bi_{UPD} and Pb_{UPD} 28
  5-2. Note on the adsorption scheme of $HO_2C(CH_2)_nCO_2H$ for the G_{EME}–$tilt_{mol}$ modeling 28
  5-3. Extension of the θ_{orb}–$t_{eld\text{-}head}$ framework to FCC-hollow adsorption of thiolates on Au(111) 29

Glossary 31

References 32

1. Preparation and Characterizations of Bi_{UPD} and Pb_{UPD} Bimetallic Electrodes.

1-1. Preparation of Bi_{UPD} and Pb_{UPD}

Bare Au electrodes were prepared by thermally evaporating a 100-nm-thick Au film on glass slides, pre-deposited with a 5-nm-thick chromium adhesive layer. Prior to metal deposition, the slides were cleaned using a piranha solution, a 3:1 (v/v) mixture of 98% H_2SO_4(*aq*) and 30% H_2O_2(*aq*). *Caution: Piranha solution reacts exothermically and violently with organic compounds, and must be handled with extreme care.*

A single-atom-thick adlayer of bismuth or lead was then deposited electrochemically onto the bare gold surface *via* underpotential deposition (UPD)[1,2] and is hereafter referred to as Bi_{UPD} or Pb_{UPD}.

Figure S1 presents cyclic voltammograms (CVs; CHI 460, CH Instruments, Austin, TX, USA) for Bi_{UPD} and Pb_{UPD} formation. These curves are consistent with previously reported voltammograms[3-8] for gold electrodes with a preferential (111) orientation. For measurements of single-molecule junction conductance, the gold electrodes were potentiostatted for 10 seconds at potentials positive of the respective bulk deposition potentials, at approximately +0.13 V (*vs.* $E_{Ag/AgCl}$) for Bi_{UPD} and −0.27 V for Pb_{UPD}, as marked by the arrows in Figure S1. After deposition, the electrodes were emersed under potential control, rinsed thoroughly with ethanol, and dried under a stream of N_2 gas.

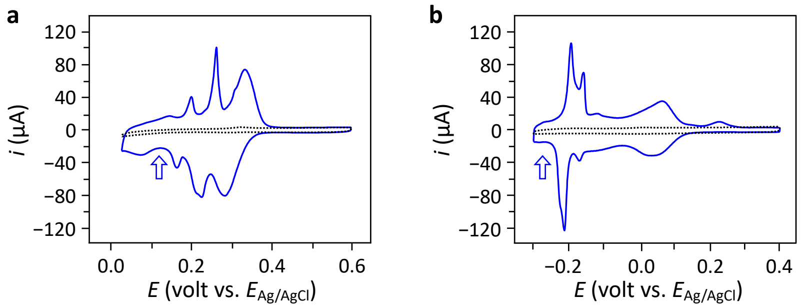

Figure S1. Deposition potentials for the preparation of UPD-modified electrodes. Cyclic voltammograms of (a) Bi_{UPD} and (b) Pb_{UPD} on gold electrodes. Arrows indicate the potentials used for monolayer deposition. Black dotted traces represent background scans in 0.1 M $HClO_4$(*aq*). All solutions were purged with N_2(*g*) to remove dissolved O_2. Conditions: Supporting electrolyte: 0.1 M $HClO_4$(*aq*); Solutes: a, 1 mM Bi_2O_3, b, 1 mM $Pb(ClO_4)_2$; Scan rates: a, 20 mV/s, b, 50 mV/s.

1-2. Characterizations of Bi_{UPD} and Pb_{UPD} adlayers

Structural characterizations by X-ray scattering and scanning probe microscopy. Detailed atomic-level insights into Bi_{UPD} have come from investigations on preferentially (111)-textured Au electrodes using *in situ* electrochemical techniques, such as atomic force microscopy (AFM),[3,9] scanning tunneling microscopy (STM),[3] and surface X-ray scattering (SXS).[3-6] The deposition begins with disordered Bi adsorption on clean Au(111),[3-6,9] followed by the formation of a commensurate (2×2) phase at approximately 0.25 monolayer (ML) coverage.[3-6,9] At more negative potentials, this structure transforms into a uniaxially commensurate (p×√3)-Bi phase, where Bi atoms organize into zigzag rows without full lattice registry.[3-6,9] The (p×√3) structure is a close-packed monolayer before bulk deposition and is associated with a sharp voltammetric feature, signifying a phase transition.[5,6]

For Pb_{UPD} on Au(111), *in situ* AFM and STM studies revealed that Pb initially nucleates at step edges, forming islands that coalesce into a close-packed monolayer.[3,7] The resulting Pb monolayer adopts a hexagonal close-packed structure with an atom-atom spacing of 0.35 (±0.02) nm and is rotated by 2°–5° relative to the Au(111) substrate,[10] generating a distinct Moiré pattern.[7,8] SXS and *in situ* electrochemical STM confirmed that the Pb layer is incommensurate and becomes compressed with increasing cathodic potential, suggesting that lateral adatom interactions dominate structural organization.[10]

Partial charge transfer on Bi adatoms. Experimental investigations of Bi_{UPD} on gold electrodes indicate that the process involves incomplete electron transfer, with Bi adatoms retaining a partial positive charge. Early work using electrochemical quartz crystal microbalance (EQCM) on

polycrystalline gold reported an electrosorption valency of ~2.7 electrons per Bi atom, suggesting a residual charge of approximately +0.3.[11] Impedance spectroscopy studies further support this view by revealing capacitive and reversible behavior, consistent with electrostatic interactions between partially charged Bi adatoms and coadsorbed anions.[12] More recent work on Au(111) single crystals reinforces this finding: using SXS and charge integration, Tamura *et al.* determined a charge density of ~165 μC/cm² for the (p×√3)-Bi adlayer, corresponding to a partial electron transfer of 2.6–2.8 electrons per Bi atom.[4,6] Similar conclusions were drawn from nanogravimetric and stress measurements by EQCM,[13] and from monolayer coverage analysis *via* SXS and STM.[3] Collectively, these studies and a comprehensive review by Oviedo *et al.*[2] suggest that Bi adatoms are not fully reduced under UPD conditions, with a nominal residual charge of +0.2 to +0.4 prior to bulk deposition. In contrast, Pb_{UPD} on gold has been concluded no significant indication of residual partial charge at complete coverage.[14]

Characterization of Bi_{UPD} and Pb_{UPD} by X-ray photoelectron spectroscopy. UPD adlayers are generally considered less susceptible to oxidation than their bulk metallic counterparts, a behavior attributable in part to differences in electronegativity. The Pauling electronegativity values for Bi (1.9) and Pb (1.8) are lower than that of Au (2.4), suggesting that electron density is preferentially drawn toward the Au substrate, resulting in the adatoms partially electron-deficient and thus positively charged. To investigate the chemical states of Bi and Pb in the UPD monolayers, X-ray photoelectron spectroscopy (XPS; PHI Quantes, ULVAC, Japan; Al Kα radiation, 1.486 keV) was employed, with comparisons made to reference spectra of both elemental and oxidized species. Prior to measurement, sample surfaces were subjected to gentle Ar^+ sputtering (0.5 keV) to remove surface contaminants. All binding energy (BE) values were calibrated against the Au $4f_{7/2}$ peak at 84.0 eV as an internal reference.

The XPS spectrum of Bi_{UPD} (Figure S2a) reveals Bi 4f peaks that are shifted by more than 1 eV to higher BE relative to metallic Bi^0 ($4f_{5/2}$: 162.2 eV; $4f_{7/2}$: 156.9 eV),[15] yet still positioned lower than those associated with Bi_2O_3 ($4f_{5/2}$: 164.3 eV; $4f_{7/2}$: 159.0 eV).[15] This intermediate BE values support a partially oxidized chemical state. In contrast, the Pb_{UPD} spectrum (Figure S2b) exhibits more modest shifts relative to Pb^0 ($4f_{5/2}$: 141.7 eV; $4f_{7/2}$: 136.9 eV), with values that remain appreciably below those of PbO ($4f_{5/2}$: 142.2 eV; $4f_{7/2}$: 137.5 eV),[15] suggesting a less pronounced charge redistribution. These results support the conclusion that Bi_{UPD} and Pb_{UPD} adatoms exhibit a partial positive charge, consistent with prior electrochemical[11] and nanogravimetric analyses (EQCM).[13] The observed partial oxidation may underlie the enhanced resistance of these UPD adlayers to molecular oxygen under ambient conditions when compared to their bulk metallic forms.

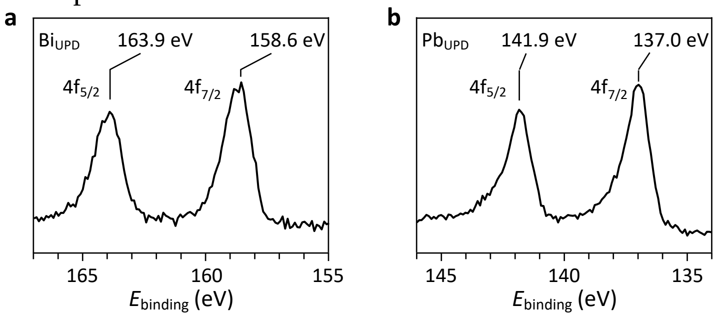

Figure S2. XPS spectra of Bi_{UPD} and Pb_{UPD} adlayers. The 4f spin-orbit doublets of (a) Bi and (b) Pb exhibit binding energies ~1.7 eV and 0.1–0.2 eV higher, respectively, than those reported for their corresponding bulk elemental forms. Compared to fully oxidized references, the Bi 4f peaks are ~0.4 eV lower than those of Bi_2O_3, and the Pb 4f peaks are 0.3–0.5 eV lower than those of PbO, supporting the assignment of a partially charged state for both UPD adlayers.

Characterization of Bi_{UPD} and Pb_{UPD} by ultra-violet photoelectron spectroscopy. Ultra-violet photoelectron spectroscopy (UPS; PHI 5000 Versa Probe, ULVAC, Japan; helium light source, HeI, 21.2 eV) was employed to characterize the surface valence bands of Bi_{UPD} and Pb_{UPD} adlayers. The sample cleaning followed the same Ar^+ sputtering procedure described in the last paragraph. Compared to pristine Au, the presence of Bi or Pb overlayer caused only slight changes in spectral shape at low binding energies (Figure S3). No additional spectral features attributable to Bi or Pb valence states were observed, consistent with the flat DOS nature of *p* bands, and the much smaller photoionization cross-section of Bi 6*p*[16] or Pb 6*p*[17] orbital compared to *d* orbitals at the incident photon energy (21.2 eV).[18]

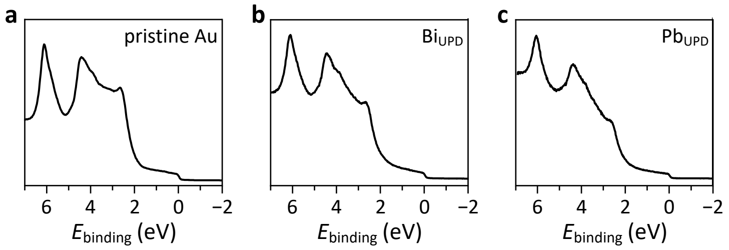

Figure S3. UPS characterization of Au and UPD-modified electrodes. UPS spectra of (a) pristine Au, (b) Bi_{UPD}, and (c) Pb_{UPD} surfaces. The spectra manifested the reduced intensity of the Au *d*-band in the low $E_{binding}$ region after Bi_{UPD} and Pb_{UPD} modification.

2. Processing and Analysis of MJM Conductance Traces

Fluctuations in the raw conductance–time (G_{raw}–t) traces arise even in the absence of bridging molecules, due to factors such as mechanical instabilities, electrical noise, or environmental perturbations. The fluctuations may originate from changes in interelectrode distances, such as diffusion of Au atoms or solvent passing the STM tip-substrate gap, as previously proposed.[19,20] Consequently, a rigorous analytical protocol is essential to reliably distinguish molecule-mediated signals from background artifacts.

We adopt the signal-processing framework by the group of Chang, detailed in the Supporting Information accompanying their *Analytical Chemistry* article.[21] It includes procedures for (i) identifying and excluding multiple-molecule junction signatures (*via* criteria including the derivative of G_{raw}–t over t, and signal to noise ratio to identify a G_{EME}), (ii) converting background tunneling conductance (G_{bkgnd}) into electrode gap distances (d_{gap}), and (iii) estimating the molecular tilt angle ($tilt_{mol}$) relative to the surface normal. Our analysis closely follows this established methodology, with one notable modification: while Chang *et al*. counted only discrete conductance-jump events, we construct histograms from all data points within each identified single-molecule junction.

The following subsections validate our data processing methodology on benchmark systems. With these controls in place, we tabulate the results associated with the single-molecule junction conductance (G_{EME}) across three electrode materials, bare Au, Bi_{UPD}, and Pb_{UPD}, and present 2D histograms of G_{EME} *versus* $tilt_{mol}$.

2-1. Validation of data processing and results of MJM measurements

To validate our data processing protocol, we reproduced MJM measurements for $H_2N(CH_2)_6NH_2$ on Au and compared our resulting histogram (Figure S4a, upper panel) with a re-plot of the dataset reported by Chang and co-workers (Figure 1g of Ref. 21; Figure S4a, lower panel). The strong agreement between the two histograms confirms the fidelity and reproducibility of our analytical implementation.

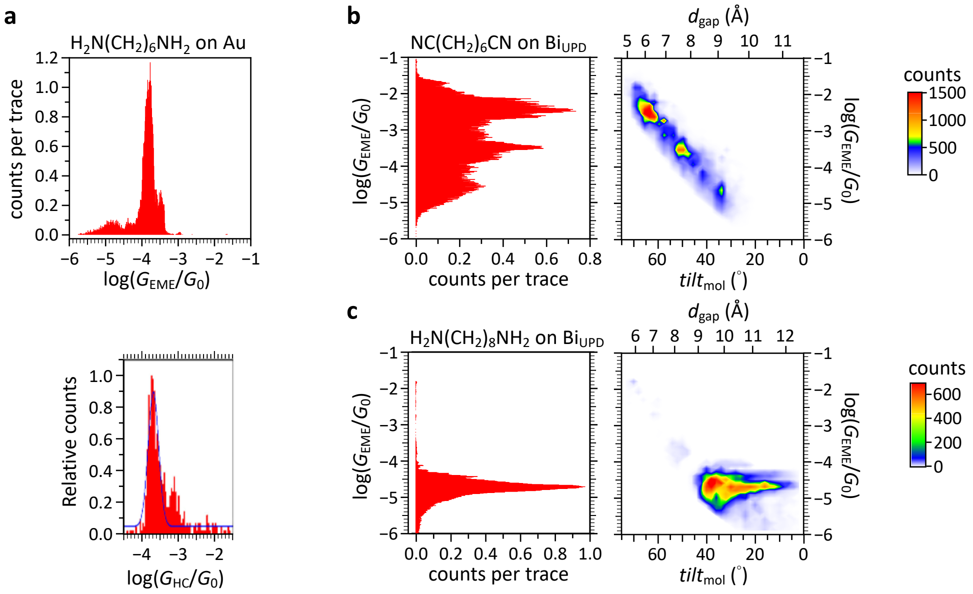

Figure S4. Validation and representative application of the MJM-based data processing protocol. (a) Conductance histograms for $H_2N(CH_2)_6NH_2$ on Au. (upper panel) This current study; (lower panel) a re-plot of the original data from Chang *et al.* (adapted from Figure 1g in Ref. 21). The two histograms show close agreement. (b) 1D and 2D histograms for $NC(CH_2)_6CN$ on Bi_{UPD} electrodes, showing a clear dependence of G_{EME} on $tilt_{mol}$, with higher conductance observed at larger $tilt_{mol}$ angles (smaller d_{gap}). (c) Corresponding histograms for $H_2N(CH_2)_8NH_2$ on Bi_{UPD}, where G_{EME} is relatively independent of $tilt_{mol}$. The absence of conductance data at small d_{gap} (< 0.8 nm) reflects dominance of background tunneling currents, which was removed during processing. Reproduced from [21]. Copyright 2020 American Chemical Society.

Panels (b) and (c) of Figure S4 display representative 1D and 2D histograms prepared from MJM-acquired G_{EME}–t traces for $NC(CH_2)_6CN$ and $H_2N(CH_2)_8NH_2$, respectively, measured on Bi_{UPD} electrodes. These two molecules exhibit markedly different G_{EME}–$tilt_{mol}$ relationships. For $NC(CH_2)_6CN$ (Figure S4b), increasing interelectrode distance (*i.e.*, a smaller $tilt_{mol}$) results in a continuous decrease in G_{EME}. Conversely, for $H_2N(CH_2)_8NH_2$ (Figure S4c), the conductance remains relatively $tilt_{mol}$-insensitive. These contrasting trends are attributed to differences in interfacial electrode-molecule hopping integrals, as discussed further in the modeling section.

The absence of conductance data at small gap distances (d_{gap} < 0.8 nm) for $H_2N(CH_2)_8NH_2$ in Figure S4c stems from the molecule-mediated current being substantially lower than the background tunneling current in this regime. As a result, even in the presence of genuine bridging events, the G_{raw}–t data appear indistinguishable from the G_{bkgnd}–t traces, and the corresponding data points are removed during background subtraction. Intriguingly, for $NC(CH_2)_6CN$ in Figure S4b the MJM approach reveals G_{EME} values significantly larger than those assigned as the high conductance set (G_{HC}) in previous break-junction (BJ) studies. This discrepancy likely originates from the snapback motion intrinsic to BJ techniques where gold electrode retraction introduces a sudden 0.5~0.7-nm gap that precludes formation of short and high-conductance junctions. In contrast, the slow, controlled motion in MJM allows the EME formation and detection of conductance at these short distances. We term these previously unobserved conductance values as the ultrahigh conductance set (G_{uHC}). Similar G_{uHC} sets are also observed for $HO_2C(CH_2)_nCO_2H$ junctions, though are absent for those with anchoring groups of $–NH_2$ and –SMe.

Figure S5 shows histograms for π-contact systems, *i.e.*, $NC(CH_2)_4CN$ and $HO_2C(CH_2)_4CO_2H$ junctions. On bare Au electrodes, the HC peak is more prominent than uHC and LC sets. In contrast, on Bi_{UPD} and Pb_{UPD} electrodes, the uHC emerges as the dominant conductance peak. Changes in the corresponding molecular tilt angle ($tilt_{mol}$) are observed alongside these shifts. Following this validation and benchmarking, we applied the same data-processing protocol to all other molecule-electrode pairs. The results are summarized in Tables S1 and S2.

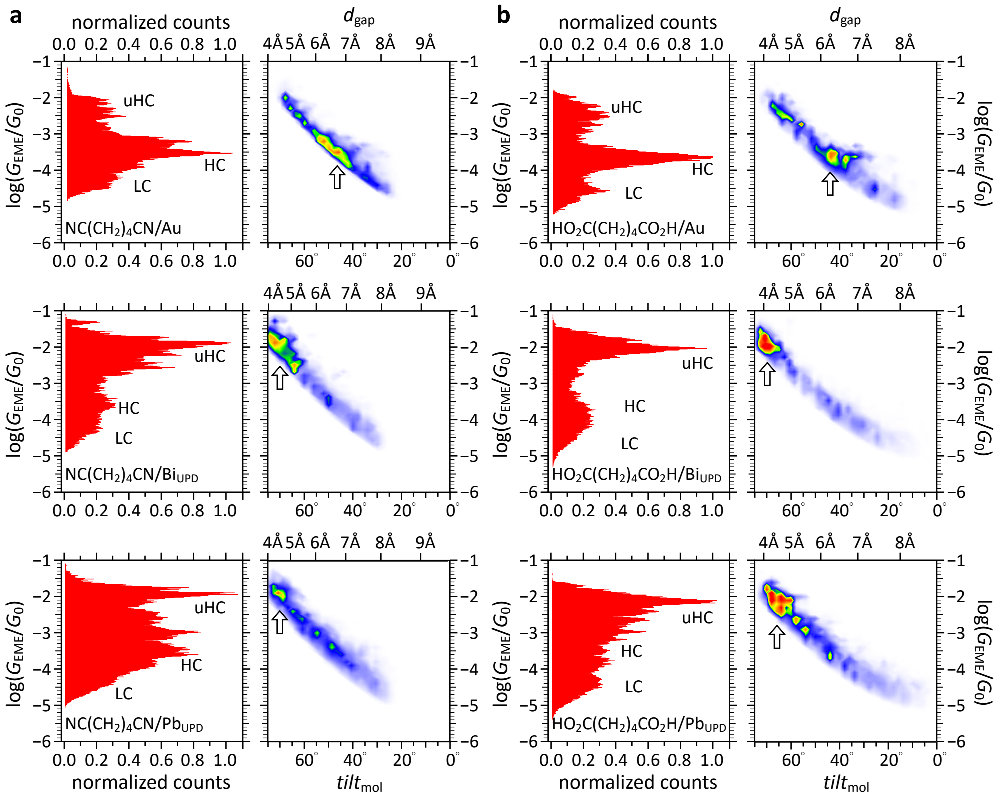

Figure S5. Electrode-dependent intensities of conductance sets. Conductance histograms for (a) $NC(CH_2)_4CN$ and (b) $HO_2C(CH_2)_4CO_2H$ measured on bare gold (top), Bi_{UPD} (middle), and Pb_{UPD} (bottom) electrodes. In the 2D histograms, arrows point toward the most probable G_{EME}. On bare Au, HC sets dominate; on UPD-modified electrodes, uHC sets predominate. Notably, uHC sets occur at d_{gap} values narrower than the typical Au snapback distance (~0.5–0.7 nm).

Table S1. Single-molecule junction conductance for α,ω-alkanes measured by MJM technique.

head group	eld	$X(CH_2)_nX$, n = 4 ($G_0 \times 10^{-4}$)			$X(CH_2)_nX$, n = 6 ($G_0 \times 10^{-4}$)			$X(CH_2)_nX$, n = 8 ($G_0 \times 10^{-5}$)			ref. no.; method
		uHC[b]	HC	LC	uHC	HC	LC	uHC	HC	LC	
$-NH_2$	Au	---	14.5	0.95	---	2.9	0.16	---	5	0.25	BJ[22]
$-NH_2$	Au	---	14.8 ±5.49	---	---	1.91 ±0.63	---	---	2.7 ±0.91	---	MJM[21]
$-NH_2$	Au	---	**11.6**[c]±1.4	0.86 ±0.27	---	**1.42** ±0.42	0.09 ±0.04	---	**2.2** ±1.3	0.2 ±0.1	this work
$-NH_2$	Bi_{UPD}	---	**13.5** ±3.8	1.19 ±0.75	---	**1.54** ±0.37	0.16 ±0.04	---	**1.7** ±0.7	0.2 ±0.1	this work
$-NH_2$	Pb_{UPD}	---	**14.5** ±5.2	1.41 ±0.42	---	**2.14** ±0.61	0.19 ±0.12	---	**2.6** ±1.4	0.3 ±0.2	this work
−SMe	Au	---	13	---	---	2.3	---	---	3.2	---	BJ[23]
−SMe	Au	---	**15.1** ±6.9	1.58 ±0.76	---	**1.88** ±0.81	0.18 ±0.10	---	**3.0** ±1.3	0.3 ±0.2	this work
−SMe	Bi_{UPD}	---	**15.2** ±8.2	1.76 ±0.28	---	**2.44** ±1.23	0.22 ±0.12	---	**3.1** ±1.4	0.4 ±0.2	this work
−SMe	Pb_{UPD}	---	**15.3** ±9.8	2.13 ±0.78	---	**1.74** ±1.00	0.28 ±0.20	---	**3.5** ±1.7	0.5 ±0.4	this work
−CN	Au	---	7.7 ±1.1	1.0 ±0.2	---	1.1 ±0.2	0.12 ±0.03	---	1.2 ±0.2	---	BJ[24]
−CN	Au	---			---	0.48 ±0.38	---	---			BJ[25]
−CN	Au	64 ±12	**5.3** ±2.0	0.51 ±0.20	15±5	**0.55** ±0.23	0.08 ±0.05	28 ±10	**1.2** ±0.5	0.2 ±0.0_3	this work
−CN	Bi_{UPD}	**107** ±36	6.4 ±1.7	0.56 ±0.28	**20**±10	1.12 ±0.57	0.11 ±0.05	**47** ±10	1.7 ±1.2	0.2 ±0.1	this work
−CN	Pb_{UPD}	**103** ±28	6.5 ±5.4	0.92 ±0.45	**25**±20	0.99 ±0.70	0.15 ±0.06	**36** ±24	1.4 ±0.7	0.3 ±0.1	this work
$-CO_2H$	Au	---	2.7	0.27	---	0.5	0.055	---	1	---	BJ[22]
$-CO_2H$	Au	55 ±14	**1.9** ±0.9	0.26 ±0.12	11±4	**0.32** ±0.18	0.05 ±0.03	16 ±8	**1.0** ±0.9	0.1 ±0.1	this work
$-CO_2H$	Bi_{UPD}	**82** ±21	4.0 ±1.9	0.45 ±0.29	**21**±14	0.91 ±0.39	0.10 ±0.04	**29** ±21	1.2 ±0.6	0.3 ±0.1	this work
$-CO_2H$	Pb_{UPD}	**69** ±26	2.2 ±1.9	0.33 ±0.22	**10**±4	0.42 ±0.38	0.11 ±0.04	**23** ±16	0.6 ±0.4	0.2 ±0.1	this work

head group	eld	$HS(CH_2)_nSH$, n = 4 ($G_0 \times 10^{-4}$)			$HS(CH_2)_nSH$, n = 6 ($G_0 \times 10^{-4}$)			$HS(CH_2)_nHS$, n = 8 ($G_0 \times 10^{-5}$)			ref. no.
		HC[b]	MC	LC	HC	MC	LC	HC	MC	LC	
−SH	Au	---	---	---	12.0	3.0[d]	---	25.0	5.0[d]	---	BJ[22]
−SH	Au	---	---	---	12.2 ±1.3	2.58 ±0.26	0.32 ±0.008	27.1 ±2.6	5.7 ±0.52	1.1 ±0.10	*i*(s)[26]
−SH	Au	---	---	---	---	---	---	21.9	4.9	1.2	BJ[20]
−SH	Au	---	---	---	12 ±2	2.2 ±0.4[d]	---	26 ±4	5.9 ±0.6[d]	---	BJ[27]
−SH	Au	---	---	---	---	---	---	25.5 ±2	5.5 ±0.5	1.2	BJ[28]
−SH	Au	95 ±31	17.6 ±9.5	1.42 ±0.39	14.6 ±8.9	2.38 ±0.99	0.29 ±0.15	21.8 ±5.0	4.0 ±2.1	0.6 ±0.2	this work
−SH	Bi_{UPD}	85 ±36	17.1 ±5.4	1.59 ±0.36	16.1 ±8.9	2.82 ±1.39	0.28 ±0.14	14.8 ±9.7	3.4 ±1.7	0.6 ±0.3	this work
−SH	Pb_{UPD}	67 ±15	11.3 ±2.4	1.26 ±0.56	14.9 ±9.7	1.26 ±0.44	0.23 ±0.10	15.6 ±4.5	2.7 ±0.1	0.5 ±0.2	this work

[a.] Most-probable G_{EME} peak values were obtained by Gaussian fits to linearly binned conductance histograms. For visualization, the manuscript presents 1D log-scaled histograms spanning six orders of magnitude; unequal bin widths in log binning distort peak shapes and bias peak positions approximately 15% higher than those of linear binning.[29-31] Therefore, the tabulated values are slightly lower than peaks observed in log-scaled plots.

[b.] Conductance sets: uHC: ultrahigh; HC: high; MC: medium; LC: low; ---: not reported.

[c.] Boldface indicates the dominant conductance set in counts-per-trace distributions (see Figure S5).

[d.] These values are classified as MC here but were reported as LC in the cited literatures.

Table S2. Contact conductance and tunneling decay constant for α,ω-alkanes by MJM technique.

head group	eld	uHC		HC		LC		ref. no.; method
		$G_{contact}$ (G_0)	β (per CH_2)	$G_{contact}$ (G_0)	β (per CH_2)	$G_{contact}$ (× 10^{-3} G_0)	β (per CH_2)	
$-NH_2$	Au	---	---	0.037 ±0.004	0.81 ±0.01	3.2 ±0.06	0.88±0.003	BJ[22]
$-NH_2$	Au	---	---	0.0742	0.99	---	---	MJM[21]
$-NH_2$	Au	---	---	0.071 ±0.009	1.03[b] ±0.03	4.7 ±2.0	1.01 ±0.08	this work
$-NH_2$	Bi_{UPD}	---	---	0.106 ±0.004	1.09 ±0.01	9.5 ±4.2	1.07 ±0.07	this work
$-NH_2$	Pb_{UPD}	---	---	0.078 ±0.016	0.99 ±0.04	7.0 ±0.2	0.98 ±0.01	this work
−SMe	Au	---	---	0.048	0.89	---	---	BJ[23]
−SMe	Au	---	---	0.072 ±0.017	0.98 ±0.04	8.8 ±2.6	1.01 ±0.05	this work
−SMe	Bi_{UPD}	---	---	0.081 ±0.018	0.98 ±0.03	9.5 ±1.1	1.00 ±0.03	this work
−SMe	Pb_{UPD}	---	---	0.054 ±0.029	0.93 ±0.08	9.1 ±1.6	0.94 ±0.04	this work
−CN	Au	---	---	0.050 ±0.007	1.03 ±0.02	5.5 ±0.9	1.02 ±0.04	BJ[24]
−CN	Au	0.18 ±0.03	0.80±0.03	0.020 ±0.013	0.94 ±0.11	1.70 ±0.16	0.88±0.012	this work
−CN	Bi_{UPD}	0.24 ±0.03	0.78±0.02	0.023 ±0.002	0.89 ±0.02	1.90 ±0.27	0.87±0.020	this work
−CN	Pb_{UPD}	0.28 ±0.06	0.83±0.05	0.032 ±0.003	0.97 ±0.01	2.14 ±0.81	0.81 ±0.06	this work
$-CO_2H$	Au	---	---	0.007 ±0.0005	0.81 ±0.01	0.60 ±0.02	0.77±0.001	BJ[22]
$-CO_2H$	Au	0.17 ±0.04	0.86±0.04	0.0060±0.0024	0.85 ±0.09	0.47 ±0.03	0.73 ±0.03	this work
$-CO_2H$	Bi_{UPD}	0.20 ±0.06	0.80±0.06	0.0141±0.0065	0.87 ±0.07	1.16 ±0.40	0.77 ±0.05	this work
$-CO_2H$	Pb_{UPD}	0.24 ±0.09	0.90±0.07	0.0082±0.0013	0.89 ±0.02	1.02 ±0.76	0.78 ±0.11	this work

head group	eld	HC		MC		LC		ref. no.
		$G_{contact}$ (G_0)	β (per CH_2)	$G_{contact}$ (G_0)	β (per CH_2)	$G_{contact}$ (× 10^{-3} G_0)	β (per CH_2)	
−SH	Au	0.66 ±1.37	1.02±0.14	0.22[c] ±0.36	1.08[c] ±0.12	---	----	BJ[22]
−SH	Au	0.50	0.96	0.082[c]	0.94[c]	---	0.45	*i*(s)[26]
−SH	Au	0.36	0.93	0.062	0.89	1.50	0.89	mix[20,d]
−SH	Au	0.60	1.01	0.10[c]	0.98[c]	---	---	BJ[27]
−SH	Au	0.41 ±0.01	0.94±0.00$_2$	0.073 ±0.018	0.95 ±0.04	3.46 ±0.02	0.80 ±0.00$_1$	this work
−SH	Bi_{UPD}	0.45 ±0.24	0.98±0.10	0.084 ±0.017	0.97 ±0.04	4.05 ±0.55	0.81 ±0.03	this work
−SH	Pb_{UPD}	0.29 ±0.06	0.94±0.04	0.054 ±0.023	0.97 ±0.09	3.11 ±0.48	0.81 ±0.02	this work

[a.] $G_{contact}$ (*i.e.*, $G_{n=0}$) and β_n are respectively the intercept and slope of the linear fit.

[b.] Our measurement results align more closely with prior MJM measurements than with STM-BJ, likely because MJM defines and subtracts background tunneling in a manner well suited to through-molecule transport.[21]

[c.] These were defined as $G_{contact}$ and β for the LC set in the corresponding literatures.

[d.] These values were obtained *via* BJ, *i*(s), and/or *i*(t) methods, where values of each conductance set bear few differences among employed methods, although certain sets undetected by certain methods, and were pooled set by set for the linear fit.

2-2. Tunneling background, interelectrode spacing, and G_{EME}–$tilt_{\mathrm{mol}}$ heatmaps

The relationship $i_{\mathrm{bkgnd}} = G_{\mathrm{bkgnd}} \times V_{\mathrm{bias}} \propto V_{\mathrm{bias}} \times e^{-\beta d_{\mathrm{gap}}}$ links the tunneling background conductance, G_{bkgnd}, with the interelectrode distance, d_{gap}. The background tunneling decay constant β is dependent on the surface environment,[32] attributed to the change in work function[33,34] or tunneling barrier[35] because of solvent or molecular adsorption. To calibrate this relationship at V_{bias} = 50 mV for solutions containing the α,ω-alkanes in the current study, we conducted linear-curve fitting on the $\log(G_{\mathrm{raw}}/G_0)$–$t$ traces of blank solution (pure 1,3,5-trimethylbenzene) using Au, $\mathrm{Bi_{UPD}}$, and $\mathrm{Pb_{UPD}}$ electrodes, and on sample solutions of $X(CH_2)_8X$ (X = NH_2, SMe, CN, CO_2H, SH). Note that for the fitting of sample solutions, we chose G_{raw} regions with few molecular signals (G_{EME})[32], typically from $\log(G_{\mathrm{raw}}/G_0)$ = –2 to −3.5, and those G_{raw}–t traces with abundant G_{EME} were also excluded in the process, such that the fitting process is decent for calibration of G_{bkgnd}–d_{gap}. Thousands of such G_{raw}–t traces were acquired at a sampling rate of 10000 points/s. Histograms of the $\Delta\log(G/G_0)/\Delta$Å slopes were thus constructed. Gaussian fitting of these histograms yielded nominal slopes (Table S3), and these values were then used to calculate the d_{gap} corresponding to any G_{bkgnd} value in MJM experiments. The generally smaller slope (in absolute value) for sample solutions compared to blank solutions is consistent with previous literature,[32] yet the origin and comparison among different headgroups are beyond the scope of current paper.

Electron tunneling between electrodes serves as a means for estimating d_{gap}; however, the tunneling background may obscure molecular signals when G_{bkgnd} over-dominates the G_{raw}. To illustrate this effect, the experimentally derived G_{bkgnd}–d_{gap} relationship (using the slope values above) was projected onto the $tilt_{\mathrm{mol}}$ axis to generate a G_{raw}–$tilt_{\mathrm{mol}}$ map, which was then compared with the MJM-derived G_{EME}–$tilt_{\mathrm{mol}}$ map. The upper panels of Figure S6a show the raw 1D-conductance histogram and a G_{raw}–$tilt_{\mathrm{mol}}$ map for $H_2N(CH_2)_8NH_2$ on Au, before background subtraction. In the 2D conductance map the black curve is the calculated G_{bkgnd}–$tilt_{\mathrm{mol}}$ correlation. The lower panels show the same data after background subtraction. Nearly no signal could be found at $tilt_{\mathrm{mol}} > 40°$, indicating that G_{EME} was buried beneath the rapidly rising G_{bkgnd} at these narrow interelectrode gaps. Figure S6b exemplifies another type of conductance histograms before and after tunneling background correction.

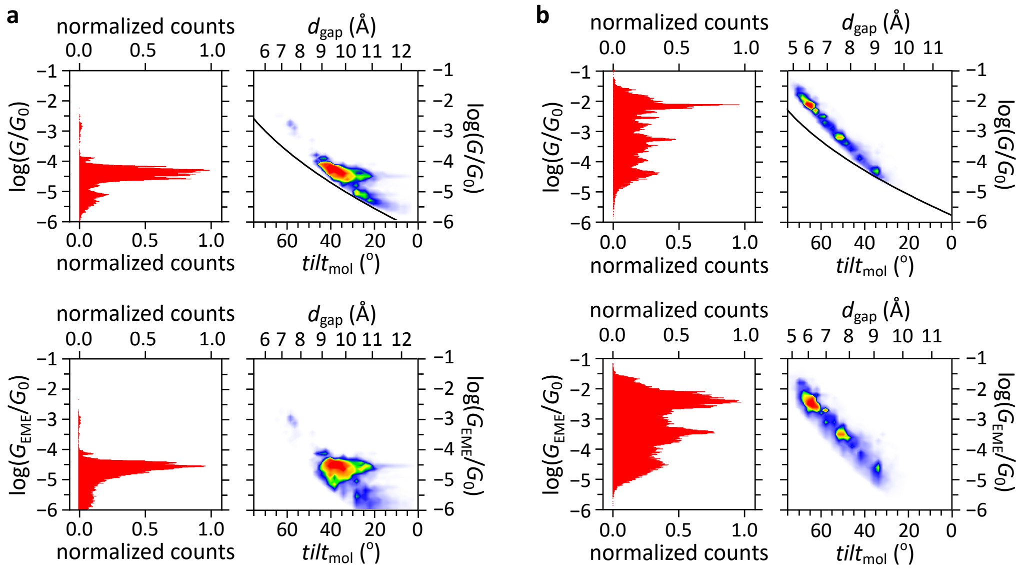

Figure S6. Background subtraction delineates single-molecule junction conductance. (a) $H_2N(CH_2)_8NH_2$ on Au and (b) $NC(CH_2)_6CN$ on $\mathrm{Bi_{UPD}}$ electrodes. (upper panels) Raw data and (lower) corresponding data after background subtraction. Black curves denote G_{bkgnd} yielded from the G_{bkgnd}–d_{gap} calibration described in this section.

Table S3. Nominal slopes of log-scale conductance-gap traces ($\Delta \log(G/G_0)/\Delta Å$) employed for G_{bkgnd}–d_{gap} calibration.

		electrode material		
		Au	Bi_{UPD}	Pb_{UPD}
headgroup	$-NH_2$	−0.411 ± 0.050	−0.404 ± 0.060	−0.400 ± 0.055
	−SMe	−0.393 ± 0.054	−0.378 ± 0.038	−0.394 ± 0.058
	−CN	−0.414 ± 0.056	−0.413 ± 0.049	−0.412 ± 0.068
	$-CO_2H$	−0.436 ± 0.060	−0.425 ± 0.056	−0.423 ± 0.049
	−SH	−0.389 ± 0.062	−0.368 ± 0.057	−0.375 ± 0.058
	TMB (solvent)	−0.514 ± 0.036	−0.447 ± 0.052	−0.447 ± 0.037

2-3. G_{EME}–$tilt_{mol}$ of $H_2N(CH_2)_8NH_2$ with clear signatures predicted by the self-derived model

This section presents G_{raw}–t traces of $H_2N(CH_2)_8NH_2$, where the measurement time (t, in seconds) reflects the progressive tip retraction and corresponding increase in d_{gap} within a single trace. As the Figure 2c2 shows, increase in G_{EME} at larger d_{gap} is observed in these traces, either continuously within a single conductance jump (*e.g.*, Figure S7a, the blue dashed box in G_{EME}–t trace for Au) or intermittently across multiple jumps (*e.g.*, Figure S7b, the blue dashed frame in the trace for Bi_{UPD}). Previous BJ measurements have suggested that the single-molecule conductance of alkane-diamine on Au is relatively insensitive to variations in adsorption geometry compared to molecules with other anchoring groups.[23] However, the trace of Figure S1 of ref. 23 exhibited a decayed conductance at shorter gap,[23] which received no exploration. Similar behavior is found in a recent article,[36] and is simply interpreted as the transition of through-solvent to through-molecule tunneling when the alkane-diamine junction is formed during gap opening. Chang and co-workers reported geometry-dependent conductance for this molecule on Au, with the conductance varying as a function of d_{gap} and $tilt_{mol}$.[21] In our study, we also observe a tilt-dependent effect, as reflected in the statistically extracted G_{EME}–$tilt_{mol}$ relationships (see Section 2-1).

***G_{EME}–$tilt_{mol}$* yielded *via* stricter statistical treatment.** Our model in Section 3-3 predicts the $tilt_{mol}$ dependence of alkane-diamine junction through the lens of headgroup-electrode coupling, assuming all-trans conformation of the alkane backbone. The similar β values reported in this work (Table S2) across electrode materials and also $tilt_{mol}$ indicates little participation of gauche-conformers in the MJM measurement, in line with our model assumption. Higher threshold of derivative of G_{raw}–t over t would yield narrower G_{EME} spread, probably thanks to nearly-total exclusion of the high-energy conformers that were reported to be less conducting than the all-trans counterpart,[26] so this "stricter criteria" were adopted herein in correspondence with the all-trans assumption for our theoretical modelling. The resulting G_{EME}–$tilt_{mol}$ in Figure S7 presents more pronounced characteristics of alkane-diamine junction than in Figure S8 and Figure S13–15. Notably, at d_{gap} ~9−10 Å or $tilt_{mol}$ ~ 40°−30°, our data reveal an increase in G_{EME} for a larger d_{gap} or a more vertical $tilt_{mol}$, strongly supporting our modelling, which interprets the G_{EME} increase by the better orbital overlap from $tilt_{mol}$ ~ 40°−30° concerning the headgroup orbital orientation (Figure S16 in Section 4-1), along with binding adjustment of nitrogen-surface atom due to adsorption-induced effect described in section 3-3-1.

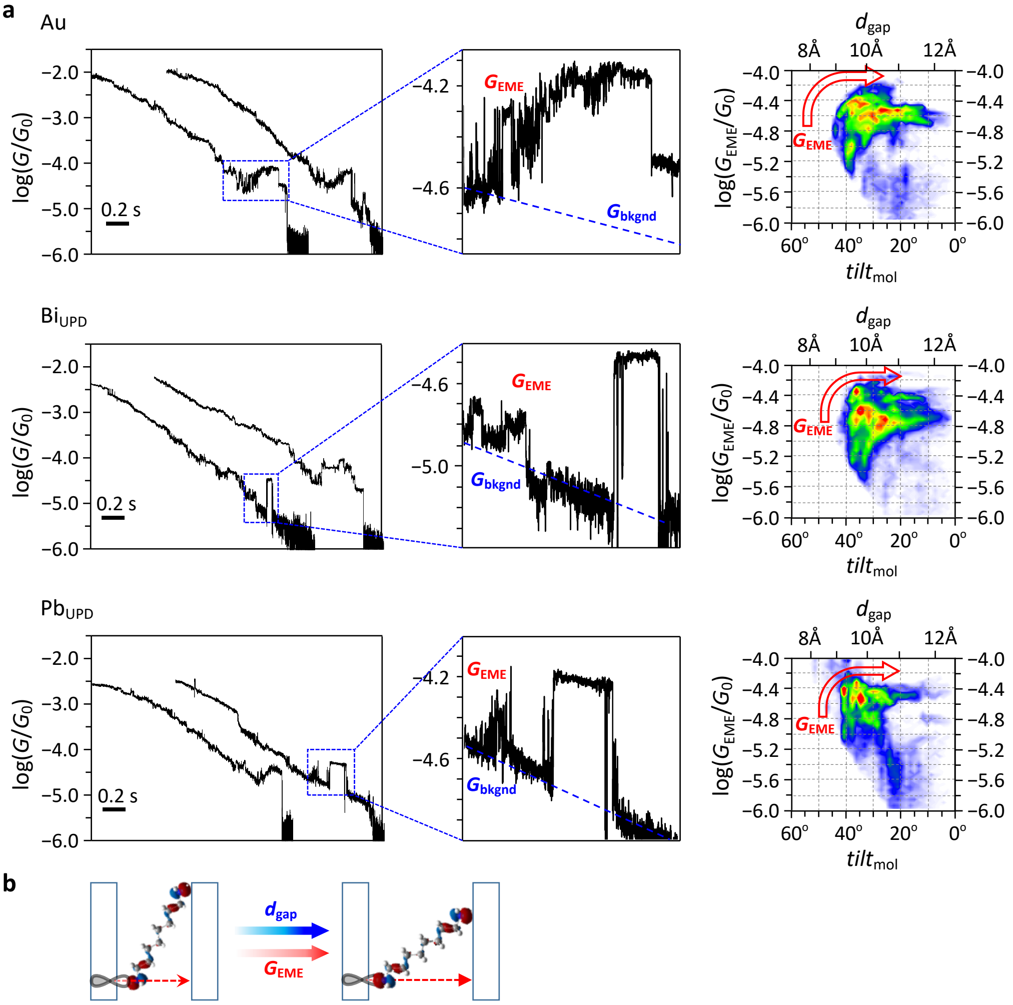

Figure S7. Conductance signatures of $H_2N(CH_2)_8NH_2$ junctions to correlate with theoretical modelling. (a) Conductance histograms measured in junctions of (upper row) Au, (medium) Bi_{UPD}, and (lower) Pb_{UPD} electrodes. (left column) Representative G_{raw}-t traces; (middle) zoom-ins highlighting the dashed blue lines representing exponentially decayed background as a measure of d_{gap}; (right) G_{EME}–$tilt_{mol}$ maps; curved arrows emphasize the increase in G_{EME} with gap opening. (b) Schematic illustrating gap opening (blue arrow); the red arrow indicates increased G_{EME} at larger d_{gap} in the intermittent conductance jumps shown in the middle panels of Figure S7a. Note these maps were yielded *via* slightly stricter statistical criteria (higher threshold of derivative of G_{raw}–t over t to identify a G_{EME}) than the others in this Supporting Information.

Comparison of *G*–gap (*G*–tilt) map signatures *via* treatments of this work and Chang *et al*. Figure S8 compares conductance histograms and *G*–gap (or tilt) maps constructed using the statistical method developed by this study and by Chang *et al.*[21] The conductance increase at d_{gap} from 8 Å to 9 Å appears more pronounced by our approach, probably because their approach defines the signal as the most probable value within a conductance jump, rather than the incorporation of all data points associated with the jump. These additional data points may encode subtle tilt-dependent features that are excluded by the peak-based approach. As to the relatively sparse signals at d_{gap} ~6–8 Å, signals appear more prominent in the treatment of Chang *et al*. than in ours, which may share the same reason just mentioned above; more specifically, their statistical treatment decrease the dominancy of the conductance sets at d_{gap} ~8–10 Å over those at shorter d_{gap}, since the data counts within the jump dwell-time were not included.

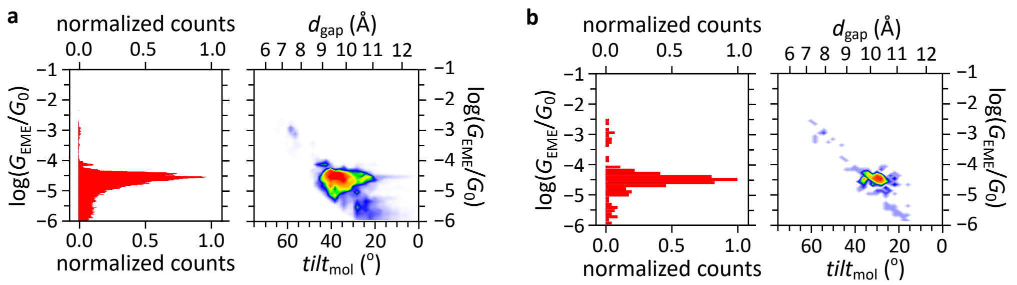

Figure S8. Conductance histograms *via* two statistical treatments. (a) This work (all-points integration within each single-molecule interval; emphasizes EME dwell time); (b) Method by Chang *et al.*[21] (one count per jump). Signals at large $tilt_{mol}$ are sparse in both approaches, the latter slightly more noticeable.

3. Theoretical Modeling and Conductance Heatmaps

In this section, we delineate a quantum transport modeling framework developed to predict how the conductance of electrode-molecule-electrode junctions (G_{EME}) depends on both the molecular electronic structure and the molecular tilt angle ($tilt_{\mathrm{mol}}$). This model integrates concepts from quantum mechanics and surface chemistry to capture the essential factors governing electron transport.

We describe molecular conductance using the Landauer formalism,[37] and formulate the electron transmission that incorporates electrode-molecule coupling by the Meir-Wingreen approach,[38] under the non-interacting limit that harnesses quantum coherence to propagating electrons. The molecule is treated as a chain of sites in the tight-binding framework, and the electrodes are modeled as semi-infinite atomic chains.[39] The conductance is calculated by evaluating electron transmission across this combined system, taking into account how molecular orbitals mix with the electrode surfaces.[15,20]

Central to our model is the *electrode-headgroup hopping integral* ($t_{\mathrm{eld\text{-}head}}$), quantifying electron propagation probability between a surface metal atom and the frontier orbital of a molecular headgroup.[40] This hopping integral depends not only on the chemical identity of the interacting atoms (described by Harrison's method for orbital overlap[41]), but also by their relative orientation in space (described *via* Slater–Koster's angular formalism[42]). This allows us to compute how junction conductance varies with $tilt_{\mathrm{mol}}$, an important geometrical variable in conductance experiments. We further account for energy-level alignment between the molecular frontier orbital and the electrode Fermi level (E_{F}) by combining surface-projected density-of-states (from DFT calculations) with Newns–Anderson–Grimley theory.[39,43,44] The result is a comprehensive conductance heatmap (G_{EME}) as a function of both molecular orbital energy and $tilt_{\mathrm{mol}}$. Presented examples include π-anchored –CN and σ-anchored $–NH_2$ headgroups, interacting with Bi_{UPD} (a representing *p*-block metal) and Au (a representing *d*-block metal) electrodes, highlighting both chemical and geometrical tunability of electron transport. These heatmaps serve as predictive guides for interpreting experimental data and designing optimized molecular-scale electronic devices.

3-1. Conductance formula for EME junctions

Electron transport across the EME junction is modeled in the coherent tunneling regime using the Landauer formalism,[37] extended by the Meir-Wingreen approach.[38] Under the low-bias experimental conditions (50 mV), the zero-bias conductance is expressed as $G = G_0 \cdot T(E)$, where $G_0 = 2e^2/h$ is the conductance quantum (77.5 μS), e is the elementary charge, h is Planck's constant, and $T(E)$ is the transmission function at an injection energy E.

In our model (Figure S9), the electrodes are treated as 1D semi-infinite atomic chains,[39] and the molecule is described by a tight-binding Hamiltonian include headgroups and a backbone.[24,45] Assuming that the molecule is perturbed by the electrodes only at the headgroup-contacting atom, the conductance at the Fermi level (E_{F}) is given by equation S1, and detailed derivations can be found in refs. 21 and 45.

$$G_{\mathrm{EME}}(E_{\mathrm{F}}) = \mathrm{G_0} \cdot \frac{\Gamma_{\mathrm{eld-head}_L} \cdot t_{\mathrm{head}_L\mathrm{-b}}}{\left(E_{\mathrm{F}} - \varepsilon_{\mathrm{head}_L}^{\mathrm{on-site}} - \Delta_{\mathrm{head}_L}\right)^2 + \frac{\Gamma_{\mathrm{eld-head}_L}^2}{4}} \cdot \frac{\Gamma_{\mathrm{eld-head}_R} \cdot t_{b\mathrm{-head}_R}}{\left(E_{\mathrm{F}} - \varepsilon_{\mathrm{head}_R}^{\mathrm{on-site}} - \Delta_{\mathrm{head}_R}\right)^2 + \frac{\Gamma_{\mathrm{eld-head}_R}^2}{4}} \cdot \frac{t_{\mathrm{head}_L\mathrm{-b}} \cdot t_{b\mathrm{-head}_R}}{t_{\mathrm{b-b}}^2} \cdot \left|\frac{t_{\mathrm{b-b}}}{E_{\mathrm{F}} - \varepsilon_{\mathrm{b}}}\right|^{2n} \quad \text{(S1)}$$

where $\varepsilon_{\mathrm{head}}^{\mathrm{on-site}}$ ($= \varepsilon_{\mathrm{head}_L}^{\mathrm{on-site}} = \varepsilon_{\mathrm{head}_R}^{\mathrm{on-site}}$) is the on-site headgroup energy, ε_{b} is the on-site energy for the backbone unit, $t_{\mathrm{head\text{-}b}}$ ($= t_{\mathrm{head}_L\mathrm{-b}} = t_{b\mathrm{-head}_R}$) is the hopping integral between the headgroup and the first (last) backbone unit, $t_{\mathrm{b\text{-}b}}$ denotes the nearest-neighbor hopping integral, $\Gamma_{\mathrm{eld\text{-}head}}$ ($= \Gamma_{\mathrm{eld\text{-}head}_L} = \Gamma_{\mathrm{eld\text{-}head}_R}$) is the electrode-headgroup coupling strength (also referred to as energy broadening), and Δ_{head} ($= \Delta_{\mathrm{head}_L} = \Delta_{\mathrm{head}_R}$) is the headgroup energy shift from $\varepsilon_{\mathrm{head}}^{\mathrm{on-site}}$ *via* the Hilbert transform of Γ.

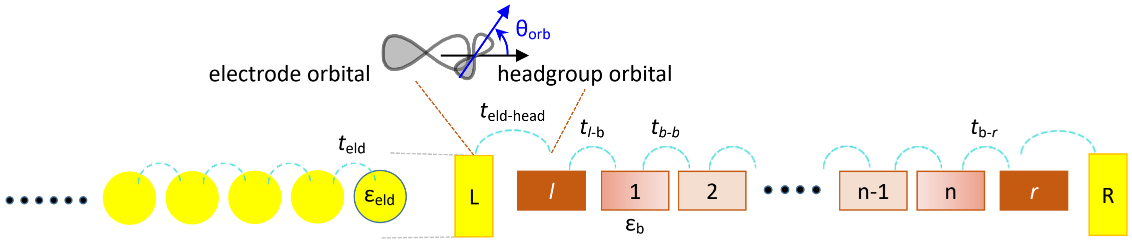

Figure S9. Tight-binding molecular junction model for deriving the interfacial propagation descriptor. Schematic illustration of a molecular junction described using a tight-binding framework, developed to extract the interfacial hopping integrals ($t_{\text{eld-head}}$) that govern electron transport across EME junctions. The left and right electrodes are modeled as semi-infinite atomic chains with orbital energy ε_{eld} and nearest-neighbor hopping integral t_{eld}. The molecule consists of two headgroup sites (head_L and head_R) and a backbone of n repeating units (sites 1 to n), each with on-site energy ε_{b}. The coupling between the headgroups and the first (last) backbone unit are denoted t_{head_L-1} ($t_{\text{n}-\text{head}_R}$), while the nearest-neighbor backbone units are coupled by $t_{\text{b-b}}$. The interfacial hopping integral $t_{\text{eld-head}}$ between the electrode and the contacting headgroup atom emphasized in this model is the key descriptor governing conductance in coherent transport through EME junctions. The upper-left drawing conceptually illustrates $t_{\text{eld-head}}$ in terms of the orbital types of the electrode and headgroup, and of their relative orientation in space (θ_{orb}).

Determination of headgroup energy shift (Δ_{head}) *via* the Newns–Anderson–Grimley method. To calculate Δ_{head} for use in Equation S1, we employed a simplified electrode model based on the Newns–Anderson formalism. In this approach, the electronic structure of the electrode is represented as a semi-elliptical density-of-states defined by two parameters: ε_{eld} (band-center) and t_{eld} (interatomic hopping integrals for adjacent electrode atoms). These parameters were obtained by fitting semi-elliptical functions to the density-of-states projected (PDOS) onto electrode surface atoms[46] from DFT calculations (Figure S10). The fit-derived band widths ($4\times t_{\text{eld}}$) and centers for the Au surface atom are consistent with the literature,[47,48] with the 6*sp* bands cutting across E_{F} at a span of ~20 eV and the narrow 5*d* bands (~5 eV) lying below E_{F} at –4 eV. For *p* metal,[49-51] surface *p* band dominates the energy range that enclosure almost all FMO of typical adsorbates. Calculation details are provided in Section 4-2. Subsequently, by employing the Newns–Anderson–Grimley framework,[30,34,35] we established the energy-level alignment between the electrode E_{F} and the isolated molecular headgroup frontier orbital. Two energy axes were defined for alignment: $y_1 = E - \varepsilon_{head}^{on-site}$ and $y_2 = \Delta_{\text{head}}$. The intersection of these axes was determined to yield Δ_{head}, calculated through the inverse Hilbert transform of $\Gamma_{\text{eld-head}}$, that is, $\Delta_{\text{head}} = \mathscr{H}(\Gamma_{\text{eld-head}})$.[39]

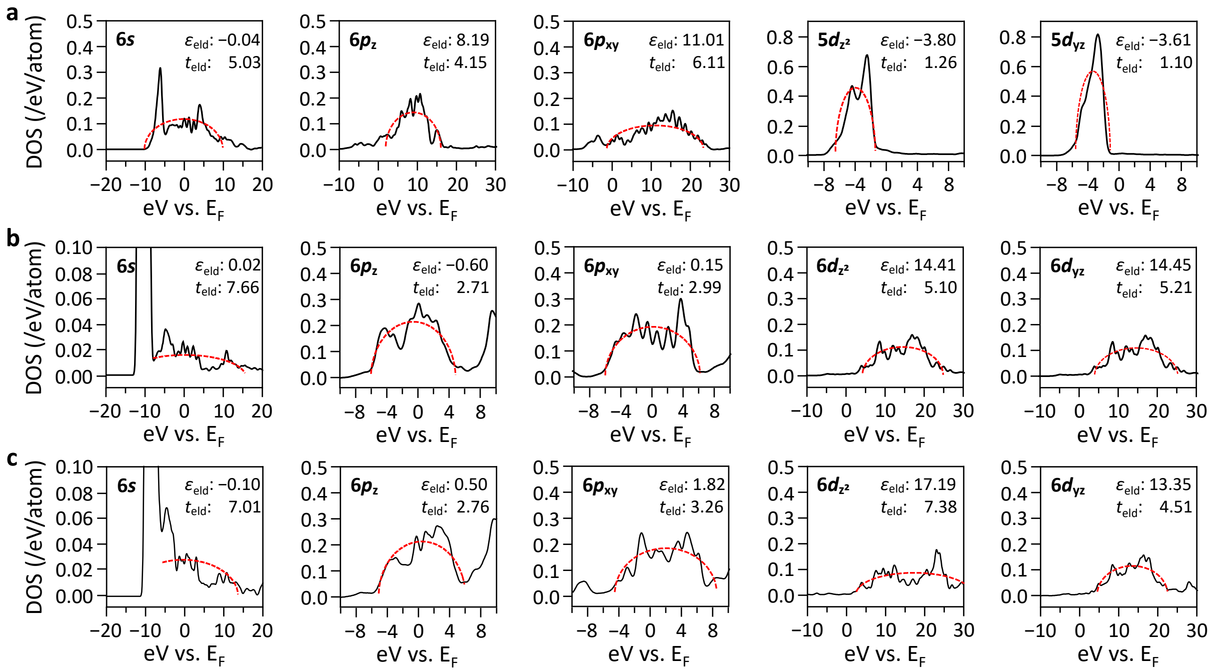

Figure S10. Calculated density-of-states projected onto surface atoms. DOS for (a) Au, (b) Bi_{UPD} or (c) Pb_{UPD} electrode surface atom. Red curves are semi-elliptical fits based on Newns–Anderson model.[39] Fitted values for the electrode band center ε_{eld} (eV *vs.* E_F) and hopping integral t_{eld} (eV) are indicated.

Relationship between $\Gamma_{eld\text{-}head}$, t_{eld}, ε_{eld}, and θ_{orb}. Under these conditions, the general expression for junction conductance simplified to a product of headgroup and backbone-related terms ($G_{EME} = G_{n=0}\cdot e^{-\beta n}$), as expressed by Equation S2.

$$G_{EME}(E_F) = G_0 \cdot \left(\frac{\Gamma_{eld\text{-}head}}{\left(E_F - \varepsilon_{FMO}^{on\text{-}site} - \Delta_{shift}\right)^2 + \frac{\Gamma_{eld\text{-}head}^2}{4}}\right)^2 \cdot \left(\frac{t_{head\text{-}b}^2}{t_{b\text{-}b}}\right)^2 \cdot e^{-\beta n} \qquad \text{(S2)}$$

Note the $\varepsilon_{head}^{on-site}$ and Δ_{head} in equation S1 is written as $\varepsilon_{FMO}^{on\text{-}site}$ and Δ_{shift} respectively, concerning the nature of α,ω-alkanes. The electrode-headgroup coupling strength, $\Gamma_{eld\text{-}head}$, depends on the electrode electronic structure and is given by the semi-elliptical expression:

$$\Gamma_{eld\text{-}head} = \frac{t_{eld\text{-}head}^2}{t_{eld}} \cdot \sqrt{1 - \left(\frac{E - \varepsilon_{eld}}{2t_{eld}}\right)^2} \qquad \text{for } -2t_{eld} < E - \varepsilon_{eld} < 2t_{eld} \qquad \text{(S3)}$$

where $t_{eld\text{-}head}$ is the electrode-headgroup hopping integral (introduced by Equation 1 in the main text), and is expressed in an angular-dependent form as $t_{eld\text{-}mol} = V\cdot\cos(\theta_{orb})$. V is the structure factor corresponding to orbital overlap. θ_{orb} is the angle between the orbitals of the contacting atoms at the electrode and the headgroup. Section 3.2 will present Harrison's method[41] for determining orbital overlap (the factor V), whereas Slater–Koster's[42] methods specifies the angular dependence ($\cos\theta_{orb}$). As to the contribution coming from the surface band, ε_{eld} and t_{eld} were obtained by fitting semi-elliptical functions to DFT-calculated PDOS on surface electrode atoms[46] (Figure S10). The derived conductance formula presented here forms the theoretical framework of our descriptor-based approach, wherein $t_{eld\text{-}head}$ emerges as a predictive and chemically interpretable metric for understanding molecule-scale electron transport.

Sensitivity to DOS parameterization. In the Newns–Anderson treatment, the electrode DOS enters the coupling Γ (imaginary part) and the level shift Δ (real part), which together determine the effective ELA used in the conductance model. For Au, Figure S10 shows a finite d-DOS near E_{Fermi} (~0.15 eV^{-1}) in addition to the *sp* contribution (~0.23 eV^{-1}). Because Γ scales with the DOS, this would increase Γ by a factor of [(0.23+0.15)/0.23] ~1.65, corresponding to an off-resonant conductance increase of ~2.7 times. In addition, reasonable uncertainties in the fitted band center/width translate

to modest variations in Δ (and thus ELA), on the order of ~0.04–0.2 eV (for details, see Section 6-7 of the Supporting Information of reference 44). These effects mainly rescale the absolute conductance but do not alter the qualitative heatmap landscape or the tilt-dependent trends.

3-2. Hopping integral $t_{\text{eld-head}}$ derived from Harrison′s and Slater–Koster′s method

The essence of $G_{\text{EME}}(E_F)$ is described by the electrode-headgroup hopping integral $t_{\text{eld-head}}$ which characterizes $\Gamma_{\text{eld-head}}$ in Equation S3 and thus enables the computation of $G_{\text{EME}}(E_F)$ using Equations S1 and S2. This subsection outlines how $t_{\text{eld-head}}$ depicted in Figure S9 is derived based on the orbital characteristics of the electrode surface and the contacting headgroup atom within a tight-binding framework.

3-2-1. Hopping integral for adsorption on *p*- and *d*-block metal electrodes: Harrison′s method.

We adopt the approach developed by Harrison and Froyen,[41,52] in which the hopping integral between two atomic orbitals is described as a function of their internuclear distance d_{nn} (in Å) and orbital angular momenta. As shown in Equation S4, the hopping integral is governed by a structural coefficient η, which reflects the orbital pairing geometry (see Table S4), and captures the extent of orbital overlap at a given distance.

This derivation leverages the resemblance between electronic band structures by nearest-neighbor LCAO (atom-based) and nearly-free-electron (NFE, plane-wave based) methods. The notations for hopping integrals $t_{\ell\ell' m(=m')}$ and structural coefficient $\eta_{\ell\ell' m(=m')}$ expressed in Harrison's handbook[41] are modified as follows:

$$t_{\text{eld}(\ell,\text{m})\text{-head}(\ell',\text{m}')} = \eta_{\text{eld}(\ell,\text{m})\text{-head}(\ell',\text{m}')} \cdot \frac{\hbar^2}{m_e \cdot d_{nn}^2}, \quad \text{for } p\text{-block metals (m (m}') = 0, 1 \text{ for } s\text{-}s, s\text{-}p, \text{ and } p\text{-}p \text{ pairs)}$$
$$= \eta_{\text{eld}(\ell,\text{m})\text{-head}(\ell',\text{m}')} \cdot \frac{\hbar^2 \cdot r_d^{3/2}}{m_e \cdot d_{nn}^{7/2}}, \quad \text{for } d\text{-block metals (m (m}') = 0, 1 \text{ for } d\text{-}s \text{ and } d\text{-}p \text{ pairs)} \qquad \text{(S4)}$$

where r_d is the *d*-state radius (in Å) for transition metals (tabulated in Harrison's handbook[41]), and m_e is the electron effective mass. The angular momentum of each orbital is given by ℓ and ℓ', while m (m') denotes the projection of the angular momentum onto the internuclear axis, following Slater and Koster's convention. Only pairings with m = m' are symmetry-allowed, and are labelled according to their bonding type: σ, π, or δ for m = 0, 1, or 2, respectively.

Table S4. Structural coefficient η of orbital pairs and bonding types.

electrode	headgroup-contacting atom											
	s				*p*							
	bonding type	$\lvert\eta\rvert$	η^4	graph	bonding type	$\lvert\eta\rvert$	η^4	graph	bonding type	$\lvert\eta\rvert$	η^4	graph
s	*ss*σ	1.40	3.84	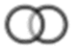	*sp*σ	1.84	11.46	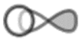	*sp*π	0	0	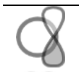
p	*ps*σ	1.84	11.46	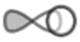	*pp*σ	3.24	110.20	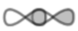	*pp*π	0.81	0.43	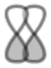
d	*ds*σ	3.16	99.71	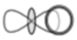	*dp*σ	2.95	75.73	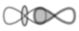	*dp*π	1.36	3.42	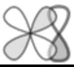

[a] Note the values of structural factors in the table are only tabulated to model the transmission functions semi-quantitatively. The larger η of *pp*σ compared to *dp*σ indicates larger extents of orbital overlap at a same inter-nuclear distance d_{nn} (in Å). Besides η, the *d*-relate hopping integrals decay faster with d_{nn} than *s* and *p* do (the power: −2 *vs*. −7/2).

[b] The values of η^4 are provided, since η enters into the transmission function in $\Gamma^2_{\mathrm{eld-head}}$.

Scaling Harrison′s structural coefficient η for chemisorbed electrode–molecule interfaces. At near-equilibrium interatomic distances in solids, band structures obtained from LCAO calculations closely resemble those from NFE models, suggesting that both atomic-like and metallic descriptions are valid in this regime. Harrison's formula for interatomic coupling typically remains applicable even when slightly deviating from equilibrium distances, although it may not hold in extreme cases where interatomic interactions are so weak that electronic states become essentially atom-like and the NFE model no longer applies.[41,52] In the case of chemisorption at an electrode–headgroup interface, as in EME junctions, DFT-calculated bond lengths are comparable to those in solids, indicating the applicability of Harrison's approach. However, because chemisorption bonds are typically weaker and involve fewer neighbors than bonds in a bulk solid, the contacting atoms experience a lower degree of orbital mixing, equivalent to a smaller structural coefficient η. Consequently, η must be appropriately scaled to achieve a realistic magnitude of the electrode–headgroup hopping integral ($t_{\mathrm{eld\text{-}head}}$), particularly for *s* and *p* orbitals.[53] Noteworthily, this scaling does not affect the G_{EME}–$tilt_{\mathrm{mol}}$ relation.

To account for the abovementioned, we scaled the structure coefficient η in our model based on the width of a frontier molecular orbital-mediated transmission peak, Γ, a well-known measure for electrode-molecule interacting strength. An example is presented in Figure S11 with transmission spectra of $NC(CH_2)_8CN$ on Bi_{UPD} electrodes. Specifically, η was scaled for the full width at half maximum (FWHM) of modelled transmission (panel a for the unscaled and panel b for the scaled one) to match that of the DFT-calculated spectrum (Figure S11c). The scaling was carried out in a self-consistent manner since the modelled transmission used DFT-optimized parameters including d_{nn}, $tilt_{\mathrm{mol}}$, and surface bands, all of which are exactly the same setups for the NEGF-DFT-transmission. The resulting FWHM (~0.52 eV, Figure S11b) was obtained as η becomes 0.417-fold of Harrison's original value (Table S4).

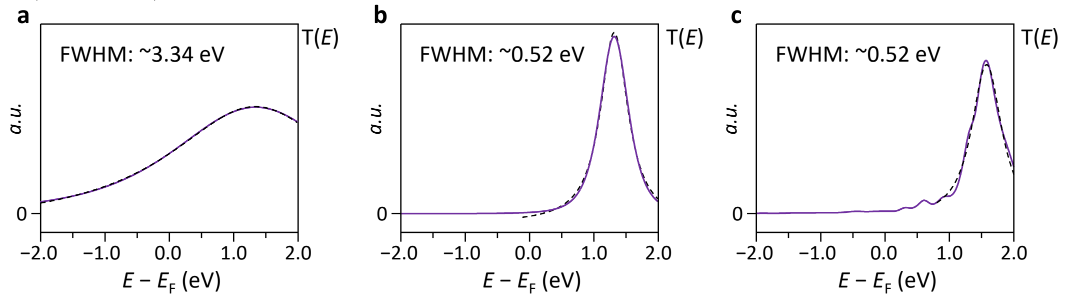

Figure S11. Scaling the structural coefficient η by transmission-width matching. Transmission spectra for $NC(CH_2)_8CN$ on Bi_{UPD} electrodes obtained by (a,b) tight-binding modeling and (c) NEGF-DFT calculations. (a) Tight-binding model using Harrison's original hopping parameters. (b) Same model with the structural coefficient scaled to η = 0.417x to match the LUMO-mediated transmission peak width (FWHM ~0.52 eV) obtained in panel c. (c) NEGF-DFT reference spectrum. Modeling input, d_{nn}, $tilt_{\mathrm{mol}}$, and electronic structures of surface band were taken from DFT-optimized interfaces. See Table S4 for η values.

The adjusted *s*/*p* hopping integrals are quantitatively comparable to those observed in oxygen adsorption on alloy metals, benchmarked against the empirical adsorption energy.[53] In contrast, no scaling was applied to *d*-*s* and *d*-*p* hopping integrals because the localized nature of *d* orbitals inherently limits their spatial extent, rendering the Harrison-based estimates consistent with prior literature.[54]

3-2-2. Angular dependence of hopping integral: Slater–Koster′s method.

The graphical illustrations in Table S4 show that Harrison′s structural coefficient η is defined for two atomic orbital axes aligned in parallel. Such a relative orientation is essentially a special case with the orbitals of the contacting electrode and headgroup atoms ($|\varphi_{\mathrm{eld}}\rangle$ and $|\varphi_{\mathrm{head}}\rangle$) are both oriented along the surface normal. To build a more general model, the hopping integral $t_{\mathrm{eld\text{-}head}}$ includes the orbital angle θ_{orb} between $|\varphi_{\mathrm{eld}}\rangle$ and $|\varphi_{\mathrm{head}}\rangle$ by applying Slater–Koster′s angular formalism[42] to the EME configurations.

In Slater–Koster′s approach, we choose a coordinate system where the *z*-axis lies along the internuclear vector $\vec{d}_{\mathrm{nn}}$ (connecting the electrode atom to the contacting headgroup atom). We then project each atomic orbital onto σ and π components defined with respect to this axis. Equivalently, each orbital is expressed as a linear combination of a σ-type basis function (aligned along $\vec{d}_{\mathrm{nn}}$) and two orthogonal π-type basis functions (perpendicular to $\vec{d}_{\mathrm{nn}}$). The coefficients in this expansion are the directional cosines of the orbital′s orientation relative to the axis, which quantify how the hopping integral changes as the orbital is tilted away from perfect alignment.

As an example, consider an *s*-*p* orbital pair: an *s* orbital on the electrode ($|\varphi_{\mathrm{eld}}\rangle$) coupling with a *p* orbital on the headgroup ($|\varphi_{\mathrm{head}}\rangle$). For simplicity, we assume the *p* orbital lies in the *yz*-plane and is tilted by an angle α from the *z*-axis (with γ fixed at π/2 to define the plane of rotation). We can decompose $|\varphi_{\mathrm{head}}\rangle$ into σ and π components aligned with $\vec{d}_{\mathrm{nn}}$, and similarly decompose the *s* orbital (which, being spherically symmetric, contributes only a σ component along the axis). This decomposition is given in Equation S5:

$$|\varphi_{\mathrm{head}}\rangle = \cos\alpha|\varphi_{p\sigma}\rangle + \cos\beta|\varphi_{p\pi+}\rangle + \cos(\gamma = \pi/2)|\varphi_{p\pi-}\rangle$$
$$|\varphi_{\mathrm{eld}}\rangle = \cos(\delta = 0)|\varphi_{s\sigma}\rangle \tag{S5}$$

where $|\varphi_{p\sigma}\rangle$ denotes the *p* orbital oriented along the internuclear axis (σ-bonding direction), while $|\varphi_{p\pi+}\rangle$ and $|\varphi_{p\pi-}\rangle$ are the two perpendicular *p* orbitals (π-oriented). $|\varphi_{s\sigma}\rangle$ is the *s* orbital′s "σ-component" (since an *s* orbital has no directional preference, it fully aligns with the axis). The hopping integral $t_{\mathrm{eld\text{-}head}}$ between these orbitals can be written as the Hamiltonian matrix element $\langle\varphi_{\mathrm{eld}}|H|\varphi_{\mathrm{head}}\rangle$. Substituting the expanded forms above, we obtain:

$$t_{\mathrm{eld\text{-}head}}(\alpha, \beta, \gamma, \delta) = \cos\alpha\cdot\cos(\delta = 0)\langle\varphi_{s\sigma}|H|\varphi_{p\sigma}\rangle + \cos\beta\cdot\cos(\delta = 0)\langle\varphi_{s\sigma}|H|\varphi_{p\pi+}\rangle + \cos(\gamma = \tfrac{\pi}{2})\cdot\cos(\delta = 0)\,\langle\varphi_{s\sigma}|H|\varphi_{p\pi-}\rangle$$
$$= \cos\alpha\cdot\eta_{sp\sigma}\cdot\frac{\hbar^2}{m_e\cdot d_{nn}^2} + \cos(\tfrac{\pi}{2} - \alpha)\cdot 0 + 0\cdot 0$$
$$= \cos\alpha\cdot\eta_{sp\sigma}\cdot\frac{\hbar^2}{m_e\cdot d_{nn}^2} \tag{S6}$$

In the second line of Equation S6 for the σ-type overlap *via* an *s*-*p* pair, we have used Harrison′s description $\langle\varphi_{s\sigma}|H|\varphi_{p\sigma}\rangle = \eta_{sp\sigma}\cdot\frac{\hbar^2}{m_e\cdot d_{nn}^2}$ which is the base hopping strength at an interatomic distance d_{nn}. The other terms, $\langle\varphi_{s\sigma}|H|\varphi_{p\pi+}\rangle$ and $\langle\varphi_{s\sigma}|H|\varphi_{p\pi-}\rangle$, vanish because of symmetry mismatch. As a result, $t_{\mathrm{eld\text{-}head}}$ depends only on the angle α (which corresponds to θ_{orb} in the main text). The hopping is maximal at α = 0 (orbital perfectly aligned for σ bonding, giving the largest *s*-*p* overlap) and decreases as cos α for tilted orientations. Equation S7 summarizes this dependence:

$$t_{\mathrm{eld\text{-}head}}(\theta_{\mathrm{orb}}) = \cos\theta_{\mathrm{orb}}\cdot\eta_{sp\sigma}\cdot\frac{\hbar^2}{m_e\cdot d_{nn}^2}, \qquad \text{where } \frac{\hbar^2}{m_e} = 7.62\ \mathrm{eV\cdot\AA^2} \tag{S7}$$

The same concept is applicable to *p*-*p* orbital pairs. For p_z-*p* orbitals, the electrode orbital is a p_z (oriented along $\vec{d}_{\mathrm{nn}}$), angle dependence of $t_{\mathrm{eld\text{-}head}}$ bears the same $\cos\theta_{\mathrm{orb}}$ character as *s*-*p* pairs and reaches maximum when the *p* orbitals align. For both p_x-*p* and p_y-*p* pairs, $t_{\mathrm{eld\text{-}head}}$ adopts a $\sin\theta_{\mathrm{orb}}$

relation due to the phase difference between σ and π orientation such that a *p* orbital oriented transverse to the $\vec{d}_{nn}$ axis has symmetric lobes cancelled out when directly aligned but increasingly overlapped at larger off-normal angles.

For *d*-*p* pairs, it is more complicated than those of *s*-*p* or *p*-*p* pairs due to the shapes of *d* orbitals.[42] We simplify an electrode *d* orbital coupling with a headgroup *p* orbital as follows.

(1) Focus on the dominant *d* orbitals: We consider only d_{z^2}-*p* and d_{yz} (d_{xz})-*p* due to their larger degrees of overlap (gauged by *η*) than the others along the surface normal (*z* axis).

(2) Treat *d*-*p* pairs analogously to *p*-*p* coupling: We model the angular dependence of each selected *d*-*p* interaction in analogy to a *p*-*p* case. The d_{z^2}-*p* interaction is modelled based on p_z-*p*, yielding a cosθ_{orb} dependence for $t_{eld\text{-}head}$. For d_{yz} and d_{xz} which have lobes directed off-axis in the *yz* or *xz* planes, d_{yz} (d_{xz})-*p* are treated like p_{xy}-*p* pairs, giving the corresponding $t_{eld\text{-}head}$ a sinθ_{orb} dependence. This approach captures the primary orientation effect for deriving *d*-*p* hopping integrals in our system, even though a fully rigorous description of *d*-*p* or *d*-*d* interactions in a bulk solid would be more complicated.[42]

3-2-3. Adsorption-induced stick-slip-like θ_{orb} realignment and d_{nn} adjustment.

Upon adsorption, the frontier molecular orbital and the metal surface orbitals tend to reorient toward one another to improve their orbital overlap,[21,55-60] closely relevant to the coupling matrix element[39,54] (equivalent to $t_{eld\text{-}head}$ in the current manuscript). The driving force to optimize the orbital overlap accelerates or impedes the rotating motion and adjust both θ_{orb} (the angle between the molecular head orbital and the surface orbital) and d_{nn} (the spacing between the contacting atoms of the electrode and the headgroup). A larger $t_{eld\text{-}head}$ has been correlated with stronger chemisorption energies, particularly when molecular orbitals couple effectively with electrode *s* bands.[54,61] This rationale also extends to *p*-block surfaces like our case of Bi_{UPD} and Pb_{UPD}. For *d* block surfaces, the inherently repulsive interaction with low-lying *d* bands[54] can offset the coupling with the 6*s* band, reducing overall energy stabilization relative to *p*-block metals.

To quantitatively incorporate this orbital realignment into our model, we introduce angle-dependent parameters for both orbital orientation θ_{orb} and interatomic spacing d_{nn}. We define the initial orbital mismatch $\theta_{orb,initial}$ as the unadjusted orbital angle, and the final angle after realignment as $\theta_{orb,final}$. We further introduce a threshold angle $\theta_{orb,\ induct\ TH}$ to identify the onset of substantial orbital adjustment. Once this threshold is reached, the degree of orbital reorientation and interatomic spacing adjustment are described by induction rates $\gamma_{\theta_{orb}}$ and $\gamma_{d_{nn}}$, respectively. These parameters are defined as $\gamma_{\theta_{orb}} = |\theta_{orb,final} - \theta_{orb,\ induct\ TH}|/|\theta_{orb,initial} - \theta_{orb,\ induct\ TH}|$ (unitless) and $\gamma_{d_{nn}} = \Delta d_{nn}/|\theta_{orb,initial} - \theta_{orb,\ induct\ TH}|$ (Å/°). To maintain physically realistic bond lengths, we constrain the corrected d_{nn} to a lower limit of 2 Å, consistent with reasonable chemisorption distances. Additionally, the headgroup on-site energy $\varepsilon_{FMO}^{on-site}$ from Equation S2 is taken as parameter to better represent actual adsorption conditions. Ultimately, five fitting parameters ($\theta_{orb,\ induct\ TH}$, $\gamma_{\theta_{orb}}$, d_{nn}, $\gamma_{d_{nn}}$, $\varepsilon_{FMO}^{on-site}$) are optimized to reflect the realistic G_{EME}–$tilt_{mol}$ relationships observed experimentally (details presented in Section 3-3).

3-3. Conductance heatmaps: preparation and predictive modeling

This subsection describes how we construct generalized conductance heatmaps and assess their consistency with G_{EME} in representative junctions, demonstrating their potential applicability to untested systems

3-3-1. Preparation of conductance heatmaps

The conductance G_{EME} is defined in the molecule-fixed frame by the orbital orientation angle θ_{orb} (see Equations S2, S3, and S7). To construct conductance heatmaps in the laboratory frame, we transform these data in terms of the molecular tilt angle $tilt_{\mathrm{mol}}$ (the angle of the molecular backbone relative to the electrode surface normal). In practice, we apply a geometric rotation to account for the molecular backbone's orientation so that both θ_{orb} and $tilt_{\mathrm{mol}}$ can serve as horizontal axes of the conductance heatmaps.

To construct the G_{EME} heatmap as a function of $\varepsilon_{\mathrm{FMO}}^{\mathrm{on-site}}$ and $tilt_{\mathrm{mol}}$, we fit our $t_{\mathrm{eld\text{-}head}}$-based conductance model to experimental MJM data. In this fitting routine, we treat θ_{orb} and d_{nn} (from Equation S7) as adjustable parameters, explicitly including the adsorption-induced effect described in Section 3-2-3. Nonlinear curve fitting was performed in LabVIEW 2016 using the trust-region dog-leg (TRDL) algorithm with a convergence tolerance of 1×10^{-10}. Figure S12 shows representative fits for α,ω-alkane junctions (Eld-X(CH_2)$_n$X-Eld, with X = −CN, −NH_2, −CO_2H, −SMe, −SH; Eld = Au or $\mathrm{Bi_{UPD}}$). From these fits we extract five parameters: $|\theta_{\mathrm{orb,\ induct\ TH}}|$, $\gamma_{\theta_{\mathrm{orb}}}$, d_{nn}, $\gamma_{d_{\mathrm{nn}}}$, and $\varepsilon_{\mathrm{FMO}}^{\mathrm{on-site}}$. These parameters (Table S5) quantify how the frontier orbital reorients and the electrode-head atom spacing adjusts upon adsorption. The fit results reveal two key distinctions between π- and σ-type contacts. First, π-type contacts exhibit increasing G_{EME} with larger $tilt_{\mathrm{mol}}$, whereas σ-type contacts display plateau-like conductance profiles. Second, π-type contacts have larger fitted values of $\theta_{\mathrm{orb,\ induct\ TH}}$, consistent with greater angular mismatch and the need for more pronounced orbital reorientation. Despite these differences, we believe the π- and σ-type profiles could be transformed into each other roughly by taking a ~π/2 shift in the $tilt_{\mathrm{mol}}$ axis, which naturally comes from the orthogonality in their orbital symmetry. We then average the −NH_2 (σ-type) and −CN (π-type) parameters to construct generalized heatmaps, suitable for semi-quantitative description of both σ- and π-type contacts on $\mathrm{Bi_{UPD}}$ and Au electrodes (Figure S13,Figure S15). The fit parameters $\gamma_{\theta_{\mathrm{orb}}}$ and $\gamma_{d_{\mathrm{nn}}}$ for the $\mathrm{Bi_{UPD}}$ electrode are obviously larger than those of Au, probably thanks to the more attractive adsorption nature of *p*-block surfaces compared to the fully-filled *d*-block as elaborated in 3-2-3. Note the fit parameters responsible for the inducting effects of $\mathrm{Bi_{UPD}}$ and $\mathrm{Pb_{UPD}}$ are similar, indicating the attractive adsorption inherency of *p* metals.

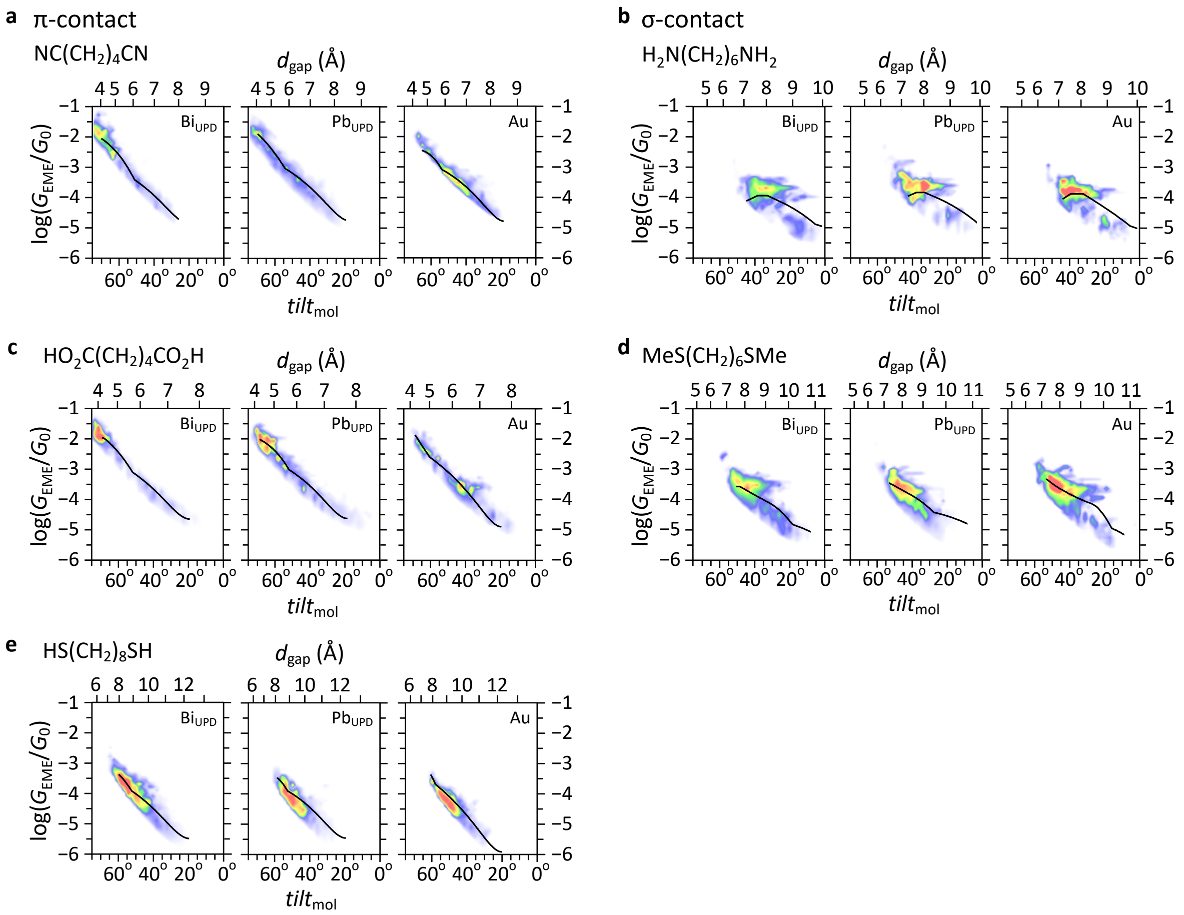

Figure S12. Nonlinear fitting with orbital inducting effect for G_{EME}–$tilt_{mol}$ profiles. (a–e) Eld–X(CH_2)$_n$X–Eld junctions with X = (a) –CN, (b) –NH_2, (c) –CO_2H, (d) –SMe, (e) –SH (Eld = Bi_{UPD}, Pb_{UPD} or Au). Black curves are best fits from the orbital-induction model (Section 3-2-3). The model optimizes five adsorption-induced parameters (see Table S5). σ-type contacts (*e.g.*, –NH_2) exhibit a roughly constant (plateau) conductance with $tilt_{mol}$, whereas π-type contacts (*e.g.*, –CN) show monotonically increasing G_{EME} with $tilt_{mol}$.

Table S5. Model fit parameters to G_{EME} – $tilt_{mol}$ conductance histograms[a].

			$\lvert\theta_{orb,\ induct\ TH}\rvert$ (°)	$\gamma_{\theta_{orb}}$	d_{nn} (Å)	$\gamma_{d_{nn}}$ (Å/°)	$\varepsilon_{FMO}^{on-site}$ (eV)[c]
σ contact	−NH_2	Bi_{UPD}	30.0	1.5	2.99	0.020	−2.44
		Pb_{UPD}	32.2	1.4	3.11	0.022	−2.13
		Au	30.1	1.3	2.65	0.013	−1.84
	−SMe	Bi_{UPD}	34.6	2.5	3.19	0.025	−1.99
		Pb_{UPD}	26.2	2.0	3.25	0.030	−1.61
		Au	37.3	3.0	2.90	0.011	−1.60
π contact	−CN	Bi_{UPD}	58.7	2.1	2.74	0.023	1.58
		Pb_{UPD}	54.7	1.7	2.73	0.025	1.40
		Au	55.5	3.0	2.60	0.012	0.73
	−CO_2H[b]	Bi_{UPD}	55.5	2.3	2.46	0.014	−1.60
		Pb_{UPD}	56.5	2.1	2.68	0.015	−1.29
		Au	49.0	1.4	2.30	0.009	−1.15
	−SH	Bi_{UPD}	57.1	2.0	2.55	0.010	−0.76
		Pb_{UPD}	57.9	2.5	2.60	0.012	−0.69
		Au	52.2	2.6	2.46	−0.0032	−0.57
Generalized G_{EME} heatmap[d]		Bi_{UPD}	44.4	1.8	2.89	0.022	−3 ~ 3
		Pb_{UPD}	43.5	1.6	2.92	0.024	−3 ~ 3
		Au	42.8	2.2	2.63	0.013	−3 ~ 3

[a] The parameters are fitted from conductance histograms of $X(CH_2)_nX$ with n = 6 for X = −NH_2, −SMe, n = 4 for X = −CN, −CO_2H, and n = 8 for −SH.
[b] For CO_2H, the complicated binding geometry should be considered, and is briefly discussed in Section 5.
[c] $\varepsilon_{FMO}^{on-site}$ is referenced to the electrode E_F and is sensitive to electrode-headgroup interaction.[33,34,62,63]
[d] Generalized heatmap parameters are averages of those for −NH_2 (6 methylene units) and −CN (4 methylene).

3-3-2. Prediction capability of the conductance heatmaps for electron transport.

Using the parameters from Table S5, we build three generalized G_{EME} heatmaps (panels (a) of Figure S13–15) to model p-block (Bi_{UPD} and Pb_{UPD}) and d-block (Au) electrodes and to serve as descriptive and potentially predictive models for interfacial electron transport. To demonstrate the application of these heatmaps (also shown as Figure 3 in the main text), we present homologous α,ω-alkanes with five representative headgroups –CN, –CO_2H, –SH, –SMe, and –NH_2, along with a series of backbone repeating units n = 4, 6, and 8.

Generalized heatmap applied to α,ω-alkanes. Note that the three generalized heatmaps are prepared from parameters averaged from those derived from $H_2N(CH_2)_6NH_2$ and $NC(CH_2)_4CN$, respectively on Bi_{UPD}, Pb_{UPD}, and Au electrode surfaces. We chose other molecules to examine the applicability of the generalized heatmaps. In the heatmap for Bi_{UPD}, there are five horizontal lines corresponding to five headgroup types (–CN, –CO_2H, –SH, –SMe, and –NH_2). The $\varepsilon_{FMO}^{on-site}$ positions for these horizontal lines are taken from the experiment-based parameters in Table S5 rather than DFT calculations of free-space molecules. In homologous alkanes, conductance decays roughly as $G_{EME} \propto e^{-\beta n}$ (n = number of methylene units); we include this effect by scaling each line based on $e^{-\beta n}$ for three backbones lengths (butyl, hexyl, and octyl chains).

The experimental results measured by the MJM technique are presented in the panels (b) of Figure S13–15, arranged in the order of headgroup type and number of methylene units. Note that the experimental results appear within a certain $tilt_{mol}$ range, limited by steric hindrance (large $tilt_{mol}$) and by the detection limit (small $tilt_{mol}$). Superimposed on the data are the cutaway views of the horizontal lines, namely, the corresponding model-predicted G_{EME}–$tilt_{mol}$ plots. Using these generalized alkane heatmaps, we then overlay our measured conductance data for molecules longer or

shorter than the reference (–NH2 or –CN with 6 or 4 methylene units). The close match between the horizontal lines on the heatmaps and the experimental conductance trends confirms that the model can semi-quantitatively predict trends for a range of chain lengths and headgroups.

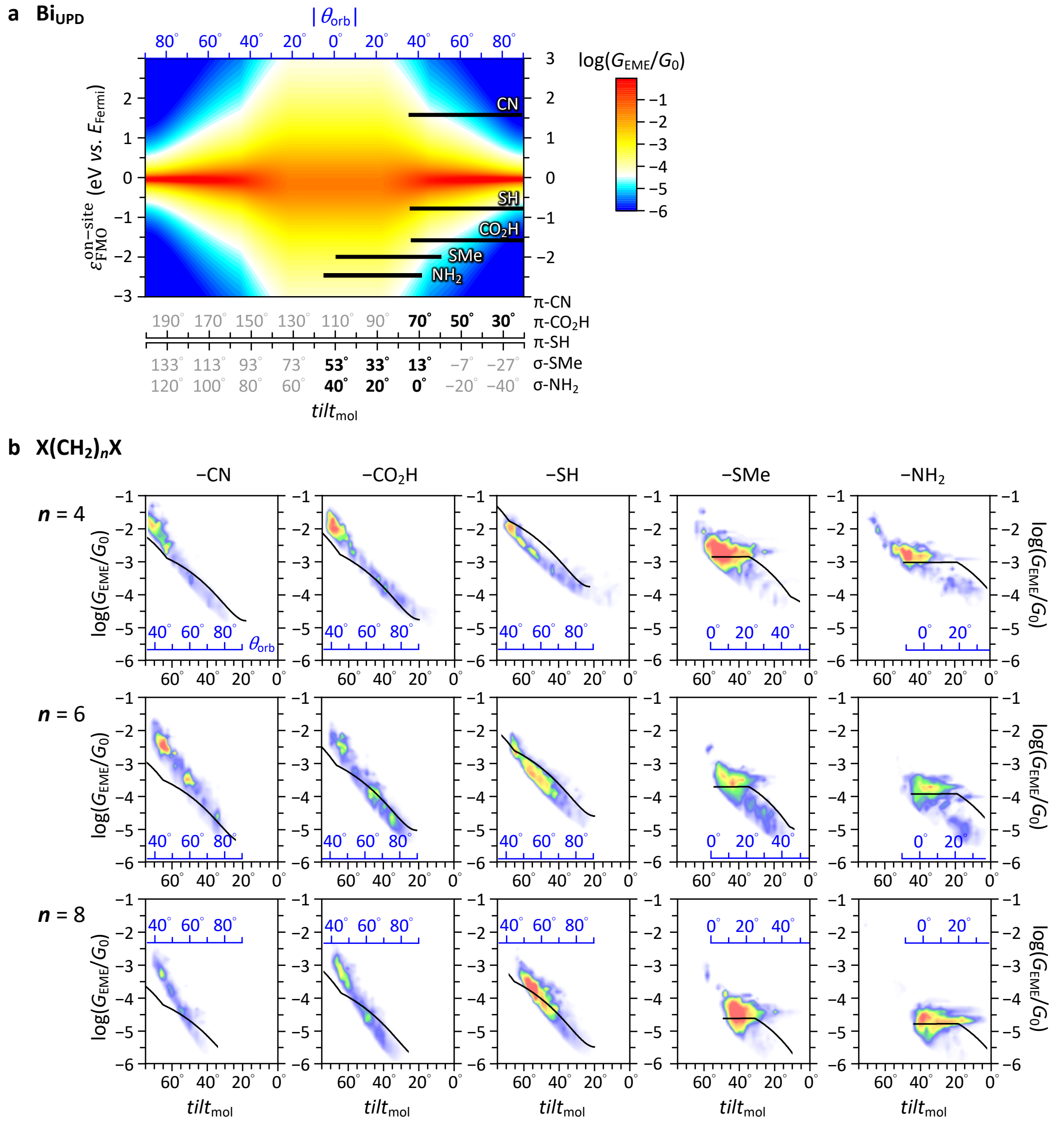

Figure S13. Generalized conductance heatmaps for representative headgroups on Bi$_{UPD}$ electrode. (a) Generalized conductance heatmaps on Bi$_{UPD}$ electrode. Heatmaps of G_{EME} as a function of orbital energy $\varepsilon_{FMO}^{on-site}$ and θ_{orb} (upper axes), also transformed to the laboratory-frame $tilt_{mol}$ (lower axes). G_{EME} heatmaps were computed using fitted induction parameters averaged from –CN (π-type) and –NH$_2$ (σ-type) headgroups. Each heatmap has five horizontal lines to exemplify molecules presented in panels b. (b) MJM experimental results and the corresponding cutaway views (black curves) from the heatmaps with headgroups at assigned $\varepsilon_{FMO}^{on-site}$ values (from Table S5) and conductance scaled by the methylene units *n*. The conductance profiles derived from the modeling generally resemble the experimental results, validating the effectiveness of the generalized conductance heatmaps.

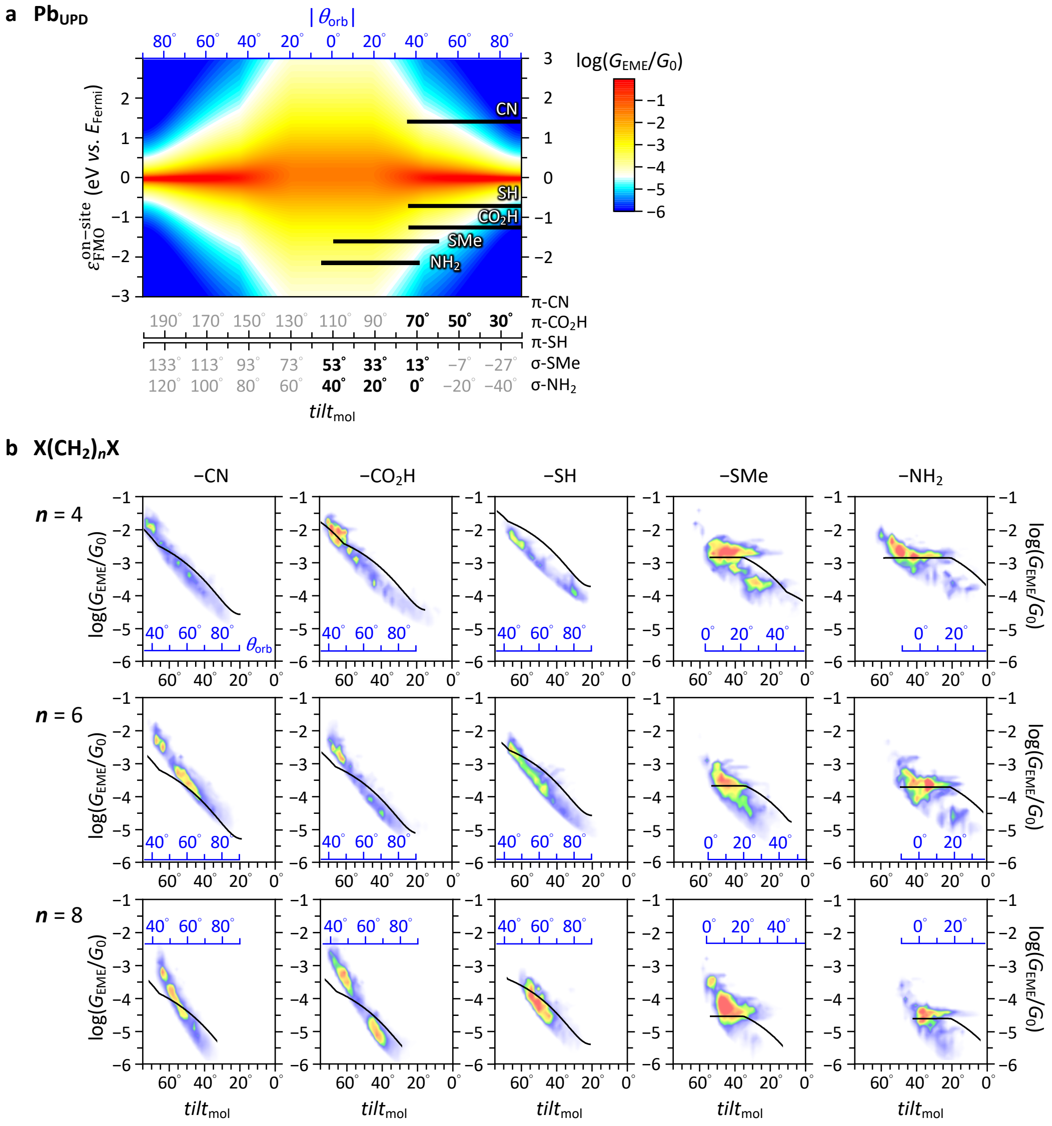

Figure S14. Generalized conductance heatmaps for representative headgroups on Pb_{UPD} electrode. (a) Generalized conductance heatmaps on Pb_{UPD} electrode. Heatmaps of G_{EME} as a function of orbital energy $\varepsilon_{FMO}^{on-site}$ and θ_{orb} (upper axes), also transformed to the laboratory-frame $tilt_{mol}$ (lower axes). G_{EME} heatmaps were computed using fitted induction parameters averaged from –CN (π-type) and –NH_2 (σ-type) headgroups. Each heatmap has five horizontal lines to exemplify molecules presented in panels b. (b) MJM experimental results and the corresponding cutaway views (black curves) from the heatmaps with headgroups at assigned $\varepsilon_{FMO}^{on-site}$ values (from Table S5) and conductance scaled by the methylene units n. The conductance profiles derived from the modeling generally resemble the experimental results, validating the effectiveness of the generalized conductance heatmaps.

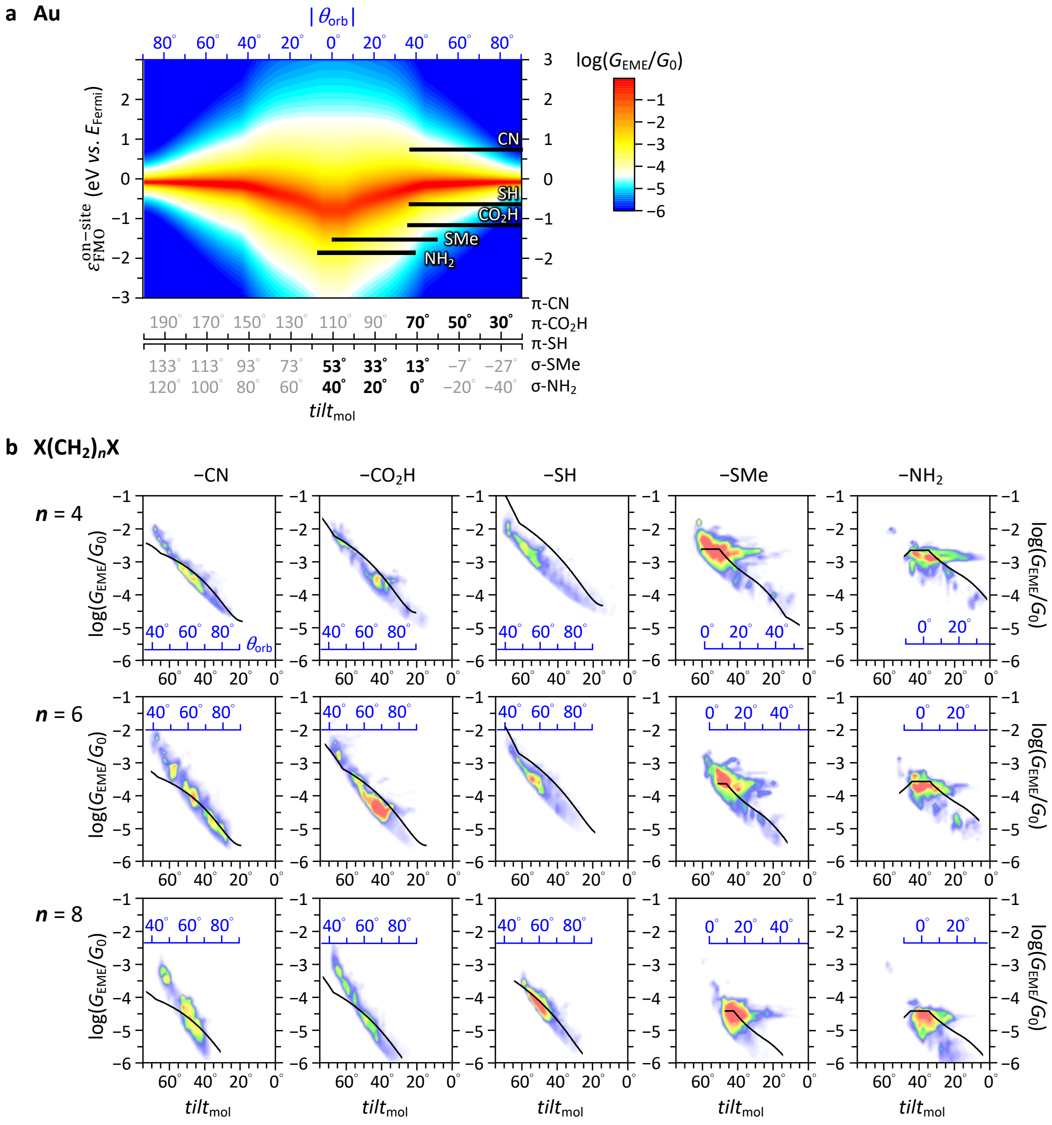

Figure S15. Generalized conductance heatmaps for representative headgroups on bare Au electrode. (a) Generalized conductance heatmaps on bare Au electrode. Heatmaps of G_{EME} as a function of orbital energy $\varepsilon_{\mathrm{FMO}}^{\mathrm{on-site}}$ and θ_{orb} (upper axes), also transformed to the laboratory-frame $tilt_{\mathrm{mol}}$ (lower axes). G_{EME} heatmaps were computed using fitted induction parameters averaged from –CN (π-type) and $–NH_2$ (σ-type) headgroups. Each heatmap has five horizontal lines to exemplify molecules presented in panels b. (b) MJM experimental results and the corresponding cutaway views (black curves) from the heatmaps with headgroups at assigned $\varepsilon_{\mathrm{FMO}}^{\mathrm{on-site}}$ values (from Table S5) and conductance scaled by the methylene units n. The conductance profiles derived from the modeling generally resemble the experimental results, validating the effectiveness of the generalized conductance heatmaps.

4. DFT and NEGF-DFT Calculations

Our $t_{\text{eld-head}}$-based model depends on the spatial distribution of FMO electron density at the molecular headgroups. Hence, this section is organized as follows. Section 4-1 visualizes gas-phase FMO of α,ω-alkanes to show that the FMO electron density is primarily localized on its headgroups. Section 4-2 details the DFT setup for electrode surfaces used to parameterize the Newns–Anderson model described in Section 3-1. Utilizing these DFT-optimized molecules and electrode surfaces, we constructed EME junctions over a series of $tilt_{\text{mol}}$ angles and calculated electron transmission through these junctions using non-equilibrium Green's function combined with DFT (NEGF-DFT).

4-1. Gas-phase FMOs and $t_{\text{eld-head}}$-based interfacial transport picture

FMOs of $X(CH_2)_8X$ (X: −NH_2, −SMe, −CN, −CO_2^-, and −S^-) were calculated in the gas-phase using Gaussian 09 at the B3LYP/6-31G(g) level. With isosurfaces depicted at an isovalue of 0.050, Figure S16 manifests that the FMO electron density of each molecule is predominantly localized on its terminal headgroups. The symmetry of this orbital density with respect to the surface normal can be categorized as π- or σ-type,[64] providing the physical basis for the $t_{\text{eld-head}}$ description of interfacial coupling.

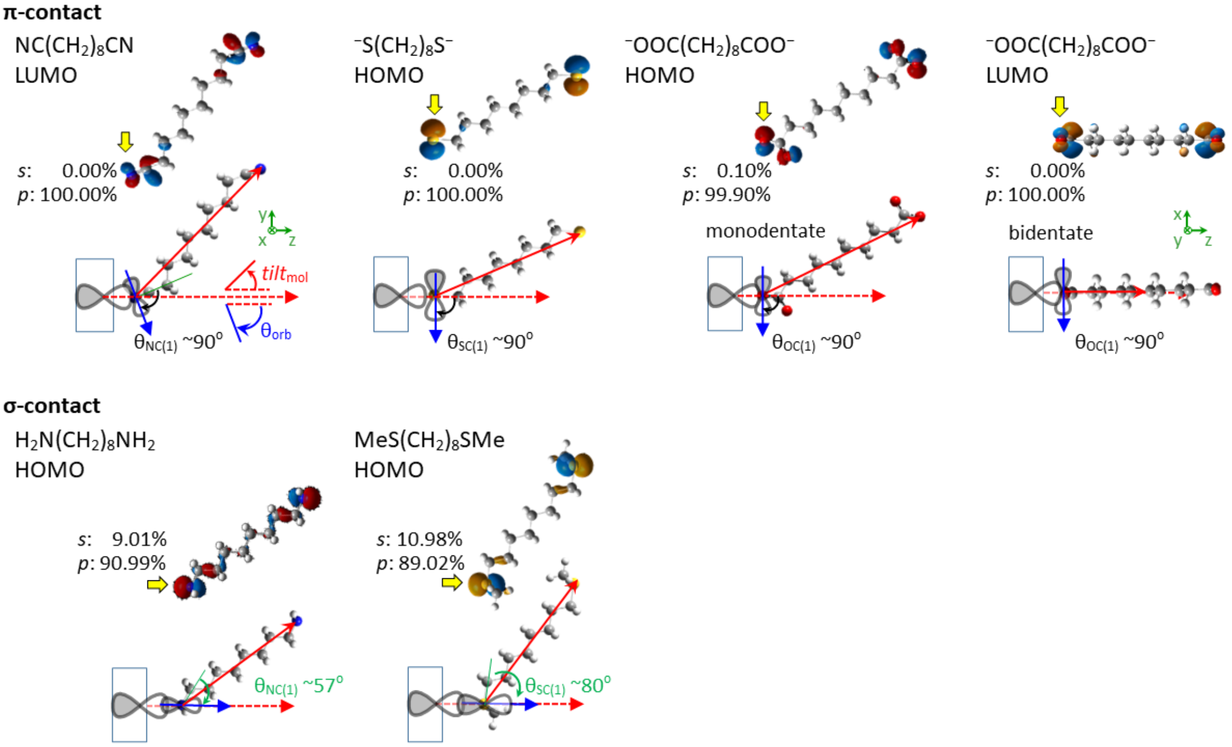

Figure S16. FMOs and interfacial-coupling schematics. For both π- and σ-contact systems, the upper panels show gas-phase FMO isosurfaces (yellow arrows point to regions most relevant to interfacial transport, and the adjacent percentages report the *s* and *p* contributions to the FMO on the terminal headgroup atom). The lower panels depict the interfacial electronic coupling between orbitals of an electrode atom and the terminal headgroup atom. For example, an electrode p_z orbital couples to a headgroup *p*-type orbital: for −CN, a half-lobe of the π* orbital; for −NH_2 or −SMe, a sp^3 lone-pair orbital (approximated as *p*-type due to its directionality). The structural angle $\theta_{XC(1)}$ (X = N, S, O) is defined as the angle between the orbital axis (blue arrow) and the X−C(1) bond axis (green line) where X is the contacting atom and C(1) is the first backbone carbon. This angle determines whether the headgroup interacts with the electrode surface in π or σ symmetry and maps the laboratory-frame $tilt_{\text{mol}}$ onto the orbital-orientation angle θ_{orb}. These schematics illustrate how relative orbital alignment governs electron transmission, providing the basis for the model's angular dependence of orbital overlap. Color code: H, white; C, gray; N, blue; S, yellow; O, red. Isovalue: 0.050.

4-2. EME junction setup and transmission calculations

Bare Au, Bi_{UPD}, and Pb_{UPD} surfaces were modeled as Au(111) slabs with lateral supercells of 8 × 8, 6 × 6, and 7 × 7 atoms, respectively. The height consisted of four Au(111) layers. The topmost layer was (i) 8 × 8 Au for bare Au, (ii) 4 × 6 Bi for Bi_{UPD}, or (iii) 6 × 6 Pb for Pb_{UPD}. In line with literature reports of a $(p\times\sqrt{3})$-Bi adlattice on Au(111) ,[3-6,9] this structure was adopted in the Bi_{UPD} modelling. Likewise, literature reported motif of hexagonally close-packed Pb monolayer on Au was constructed for Pb_{UPD}. During geometry optimization, all slab layers were fixed except the topmost layer. For the adsorbed molecule, only the contacting electrode atom was allowed to relax while positioned atop an electrode surface atom.

Transmission spectrum for the scaling of structural factor. The transmission spectrum of Figure S11 were computed by Atomistix Toolkit (QuantumATK S-2021.06-SP2, Synopsys) implemented with DFT and NEGF.[65,66] The calculations employed the Perdew-Burke-Ernzerhof (PBE) functional within the Generalized Gradient Approximation (GGA), a double-ζ with a polarization (DZP) basis set for all atoms. A mesh cutoff of 150 Ry, periodic boundary conditions with 3 × 3 × 100 k-point sampling, and an electronic temperature of 300 K were used. Iterative convergence criteria were set to a tolerance of 10^{-4} tolerance for the density matrix. These parameters were chosen to ensure well-converged transmission function.

5. Notes on Model Applicability and Additional Analyses

5-1. Layer-resolved PDOS assessment of Au(*d*)–Adlayer(*p*) orbital mixing Bi_{UPD} and Pb_{UPD}

To assess possible Au(*d*)–adlayer(*p*) orbital mixing, we calculated layer-resolved orbital-projected DOS (*s*/*p*/*d*) for Au(111), Bi_{UPD} on Au(111), and Pb_{UPD} on Au(111) using the same DFT setup described in Supporting Information Section 4-2. As shown in Figure S17, the surface adlayer (Bi_{UPD} or Pb_{UPD}) is dominated by *p* states near E_{Fermi}, while t he adlayer PDOS shows negligible Au *d* character, indicating limited orbital mixing from Au *d* orbitals with the *p* orbitals from the UPD adatoms. These results support our simplified interfacial model that treat Bi_{UPD} and Pb_{UPD} as *p*-character surfaces for the transport analysis in the main text.

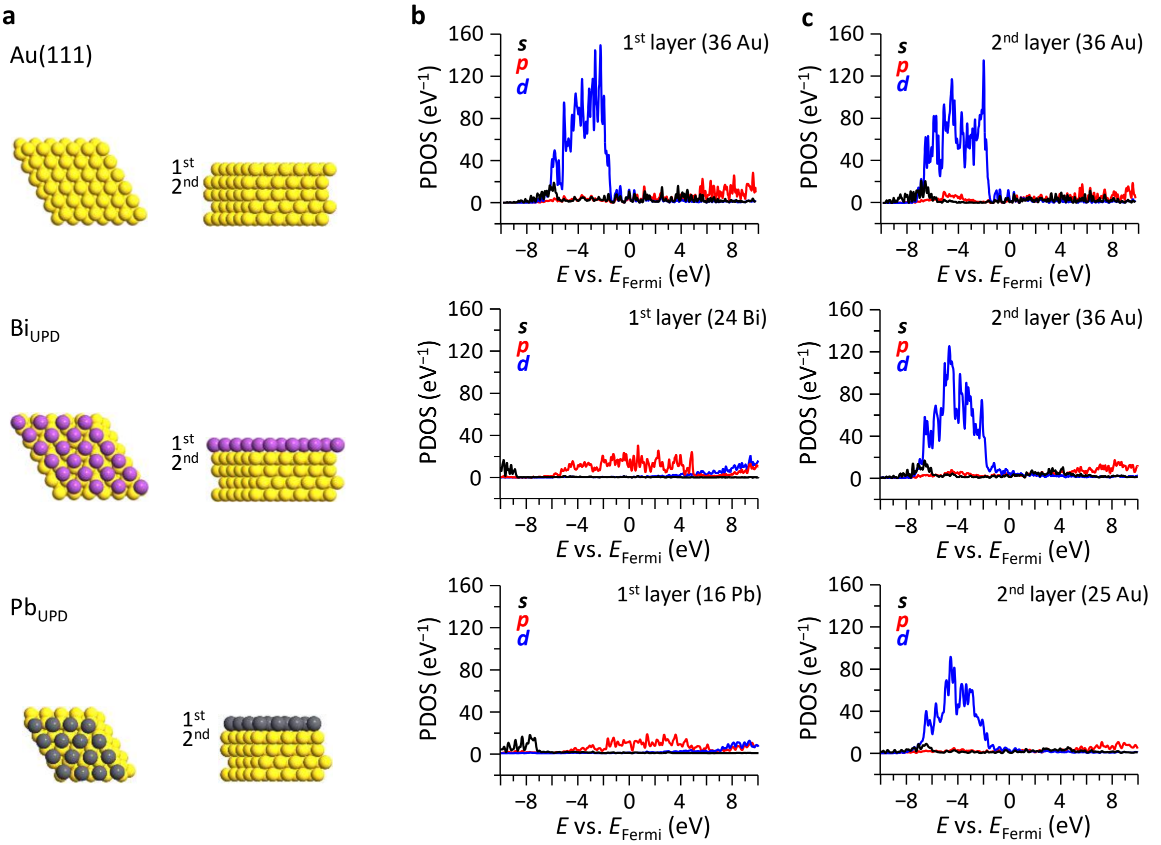

Figure S17. Layer-resolved electronic structures from DFT-calculated electrode surfaces. (a) Geometric models for Au(111), Bi_{UPD}, and Pb_{UPD}. Corresponding PDOS for the (b) first (surface) and (c) second atomic layers. In the PDOS plots, black, red, and blue curves represent *s*, *p*, and *d* contributions, respectively. For the surface layer that dominates interfacial transport, bare Au shows a pronounced *d* character, whereas the Bi and Pb adlayers exhibit dominant *p* character near E_{Fermi} with negligible *d* orbital contribution from the underlying gold layers.

5-2. Note on the adsorption scheme of $HO_2C(CH_2)_nCO_2H$ for the G_{EME}–$tilt_{mol}$ modeling

Alkanedicarboxylic acids can adopt numerous adsorption and tilt configuration in EME junctions. After surveying relevant literatures,[67-72] the most probable adsorption scheme appears to be a monodentate binding with only one oxygen atom attaching to a single metal atom, and the tilting is within its O−C−O plane (depicted in Figure S16) which is the *yz*-plane of the experimental frame. Bidentate binding configurations have been reported in many studies on alkanecarboxylic acids monolayer assembly on Ag,[67] Ag_{UPD},[68,73] and Cu_{UPD},[68,73] which confer better orbital matching of LUMO at larger tilts (Figure S16), yet the transport of alkanecarboxylic acids is known to be HOMO-mediated, derived by single-molecule transition voltage spectroscopy.[44] If we roughly model bidentate adsorption with each carboxylate oxygen positioned atop an individual Au atom, a large $\varepsilon_{\mathrm{FMO}}^{\mathrm{on-site}}$ would be feasible based on LUMO's much larger energy offset from E_F than HOMO. This results in overall conductance highlighted by the blue color scale and is about two orders of magnitude

lower than that of HOMO-mediated transmission, even improved LUMO overlap would be achieved at larger $tilt_{\mathrm{mol}}$ angles. This discrepancy strongly suggests that bidentate adsorption is unlikely to dominate the single-molecule G_{EME} measured experimentally.

In experimental setups for single-molecules junction conductance measurements utilizing STM or AFM tips, adsorption can be highly dynamic, involving possibly both monodentate and bidentate binding modes.[70,71] Clarifying such dynamic adsorption scenarios through direct surface characterization methods remains challenging and is beyond the scope of the present study.

5-3. Extension of the θ_{orb}–$t_{\mathrm{eld\text{-}head}}$ framework to FCC-hollow adsorption of thiolates on Au(111)

In this work, θ_{orb} is defined as the orientation of the anchoring atom p-like orbital axis with respect to the surface normal (Figure 1b). This definition is independent of the specific adsorption site and therefore remains applicable even when the anchoring atom interacts with multiple surface atoms. In the main text, we adopt an atop-site picture for thiolate contacts because it is the most direct realization of the Newns–Anderson treatment (adsorbate coupled to the topmost surface atom) and provides a transparent atomic-orbital (AO) overlap model to correlate θ_{orb} with $t_{\mathrm{eld\text{-}head}}$.

To address the well-established FCC-hollow adsorption geometry of thiolates on Au(111), we generalize the electrode–headgroup coupling from a single-atom contact to a three-atom contact. Specifically, we represent the electrode-side contact by a normalized linear combination of the three neighboring Au orbitals at the hollow site,

$$|\phi_{\mathrm{eff}}\rangle = \sum_{i=1}^{3} c_i \,|\phi_i\rangle \text{ with } \sum_i |c_i|^2 = 1,$$

so that the effective hopping integral entering the transport model is

$$t_{\mathrm{eff}} = \langle \phi_{\mathrm{head}}|H|\phi_{\mathrm{eff}}\rangle = \sum_{i=1}^{3} c_i t_i, \text{ where } t_i = \langle \phi_{\mathrm{head}}|H|\phi_i\rangle.$$

The angular dependence of each t_i is treated within the Slater–Koster formalism, while the distance dependence follows a Harrison-type scaling; normalization of the linear combination is performed as described by Einstein (Theodore L. Einstein, *Interactions Between Adsorbate Particles*, Elsevier: Amsterdam, 1996). This construction yields an "effective" multi-atom contact that can be used in the same conductance expression as the atop-site model, while preserving θ_{orb} as the geometric descriptor governing the orbital-overlap modulation.

Figure S18 compares the fits of the measured G_{EME}−$tilt_{\mathrm{mol}}$ trend for alkanedithiols using the atop-site model (Figure S18b$_1$) and the FCC-hollow-site model (Figure S18b$_2$). Both implementations reproduce the experimental trend well, supporting that the θ_{orb}-based description captures the dominant geometric modulation of interfacial coupling for thiolates. A visible difference between the two fits occurs at intermediate tilt angles (~20°–40°), where the FCC-hollow model predicts slightly higher conductance, consistent with an increased number of symmetry-compatible electrode orbitals contributing to the coupling when the headgroup p-like density becomes more laterally oriented.

The fitted parameters for the atop and FCC-hollow models are summarized in Figure S18c. Notably, the FCC-hollow-site fit yields a larger effective d_{nn} than the atop-site fit, consistent with literature expectations for Au–S adsorption geometries and supporting the physical reasonableness of the multi-atom contact implementation.[26] Overall, these results justify our use of θ_{orb} as a general geometric descriptor for thiolate contacts and show that hollow-site adsorption can be incorporated naturally within the present $t_{\mathrm{eld\text{-}head}}$ framework by interpreting the coupling as an effective multi-atom interaction.

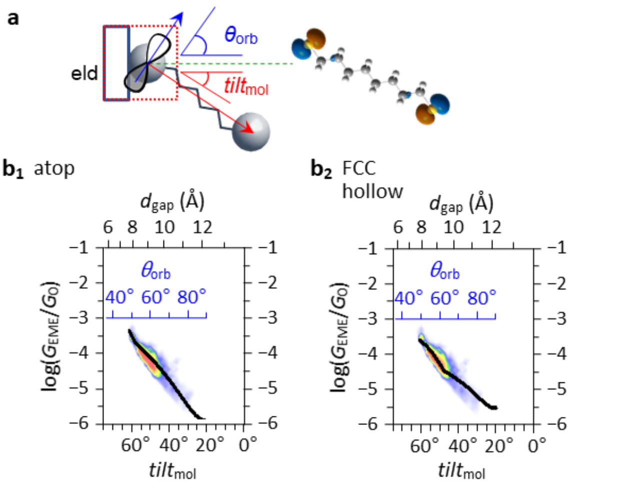

c

	$\lvert\theta_{\text{orb, induct TH}}\rvert$ (°)	$\gamma_{\theta_{\text{orb}}}$	d_{nn} (Å)	$\gamma_{d_{\text{nn}}}$ (Å/°)	$\varepsilon_{\text{FMO}}^{\text{on-site}}$ (eV)
atop	52.2	2.6	2.46	−0.0032	−0.57
FCC hollow	62.5	3.2	2.75	−0.0010	−0.67

Figure S18. Atop-site versus FCC-hollow-site implementations for thiolate contacts on Au(111). (a) Schematic illustrating that θ_{orb} is defined by the orientation of the sulfur p-like orbital relative to the surface normal, while the electrode-side coupling can involve either a single Au atom (atop) or a symmetry-adapted linear combination of three Au atoms (FCC hollow). (b) Experimental G_{EME}–$tilt_{\text{mol}}$ results for octanedithiols overlaid with the best fits from (b_1) the atop-site model and (b_2) the FCC-hollow-site model constructed from the threefold Au coordination. (c) Best-fit parameters for the atop and FCC-hollow models; the larger fitted d_{nn} for the FCC-hollow case is consistent with literature Au–S adsorption geometries.[26]

Glossary

$t_{\text{eld-mol}}$: electrode-molecule hopping integral
$t_{\text{eld-head}}$: electrode-headgroup hopping integral
t_{eld}: hopping integral for neighboring sites of the electrode
$t_{head\text{-}b}$: hopping integral between the headgroup and the first (last) backbone unit
$t_{b\text{-}b}$: hopping integral for nearest-neighbor backbone unit
$t_{\text{eld}(\ell,\text{m})\text{-head}(\ell',\text{m}')}$: hopping integral adapted from Harrison's notation; ℓ (ℓ') for angular momentum and m (m') for projection of the angular momentum onto the internuclear axis
$\eta_{\ell\ell'\text{m}(=\text{m}')}$: structural coefficient from Slater–Koster′s and Harrison's notation
r_{d}: *d*-state radius (in Å) for transition metals in Harrison's handbook
m_e: electron effective mass
$tilt_{\text{mol}}$: molecular tilt angle
$\hat{H}_{\text{eld}}$: Hamiltonian of the electrode
$\hat{H}_{\text{mol}}$: Hamiltonian of the molecule
$\hat{H}_{\text{eld-mol}}$: off-diagonal Hamiltonian for electrode-molecule interaction
$|\varphi_{\text{eld}}\rangle$, $|\varphi_{\text{mol}}\rangle$, $|\varphi_{\text{head}}\rangle$: orbital on the electrode, molecule, or headgroup
Δ_{shift}: energy shift or real part of the self-energy
Δ_{head}: headgroup energy shift or real part of the self-energy
Γ: coupling between the molecule and the electrode, imaginary part of the self-energy
$\Gamma_{\text{eld-head}}|_{E=E_{\text{Fermi}}}$: coupling between the molecular headgroup and the electrode at Fermi energy
$\mathcal{H}$: Hilbert transform
d_{nn}: interatomic distance
θ_{orb}: angle between the interfacial atomic orbitals
$\theta_{\text{orb,initial/final}}$: unadjusted/final orbital angle
$\theta_{\text{orb, induct TH}}$: threshold angle orbital angle as the onset of inducting effect
$\gamma_{\theta_{\text{orb}}}$: induction rates of orbital reorientation, $|\theta_{\text{orb,final}} - \theta_{\text{orb, induct TH}}|/|\theta_{\text{orb,initial}} - \theta_{\text{orb, induct TH}}|$
$\gamma_{d_{\text{nn}}}$: induction rates of interatomic spacing adjustment, $\Delta d_{\text{nn}}/|\theta_{\text{orb,initial}} - \theta_{\text{orb, induct TH}}|$
E_{FMO}: frontier molecular orbital level that dominates transport
E_{Fermi} (or E_{F}): Fermi energy of electrode
$\varepsilon_{\text{FMO}}^{\text{on-site}}$: on-site energy of the molecular FMO
ε_{eld}: on-site energy of the electrode (surface band center)
ε_{b}: on-site energy of the backbone unit
ELA: adsorbate energy-level alignment, $E_{\text{Fermi}} - \varepsilon_{\text{FMO}}^{\text{on-site}} - \Delta_{\text{shift}}$
MJM: molecular-junction mapping
d_{gap}: electrode gap spacing
i_{bkgnd} or G_{bkgnd}: background tunneling current or conductance
i_{EME} or G_{EME}: electrode-molecule-electrode (EME) junction current or conductance, from MJM
$G_{EME}|_{E=E_{Fermi}}$: zero-biased EME junction conductance at Fermi, in the theoretical model
G_{raw}: raw conductance obtained by MJM
$G_{\text{uHC, HC, MC, LC}}$: ultrahigh, high, medium, or low conductance set
G_0: quantum conductance, $2e^2/h$ ~77.5 μS
h or $\hbar$: Planck constant or reduced Planck constant
e: elementary charge
L_{mol}: molecular length defined as the distance between the two terminal anchoring atoms of the fully extended α,ω-alkane
n: number of methylene unit
A: prefactor for intramolecular coupling
V: orbital structure factor relevant to the extent of orbital overlap
$\Delta E_{\text{d-hyb}}$: chemisorption energy
V: metal-adsorbate coupling matrix element (equivalent to $t_{\text{eld-head}}$ in this work)
ε_{d}: energy of metal *d*-band center
ε_{ab}: energy of anti-bonding state
α: energy penalty due to orbital orthogonalization
f_d: *d*-band filling fraction

References

(1) Herrero, E.; Buller, L. J.; Abruña, H. D. Underpotential Deposition at Single Crystal Surfaces of Au, Pt, Ag and Other Materials. *Chem. Rev.* **2001**, *101*, 1897–1930.
(2) Oviedo, O. A.; Reinaudi, L.; García, S. G.; Leiva, E. P. M. *Underpotential Deposition*. Springer Cham: 2016.
(3) Chen, C.-h.; Kepler, K. D.; Gewirth, A. A.; Ocko, B. M.; Wang, J. Electrodeposited Bismuth Monolayers on Au(111) Electrodes: Comparison of Surface X-ray Scattering, Scanning Tunneling Microscopy, and Atomic Force Microscopy Lattice Structures. *J. Phys. Chem.* **1993**, *97*, 7290–7294.
(4) Tamura, K. Comparative Study of the Underpotential Deposition of Bi in an Aqueous Electrolyte and an Ionic Liquid. *J. Phys. Chem. C* **2023**, *127*, 22733–22739.
(5) Tamura, K.; Ocko, B. M.; Wang, J. X.; Adžić, R. R. Structure of Active Adlayers on Bimetallic Surfaces: Oxygen Reduction on Au(111) with Bi Adlayers. *J. Phys. Chem. B* **2002**, *106*, 3896–3901.
(6) Tamura, K.; Wang, J. X.; Adžic, R. R.; Ocko, B. M. Kinetics of Monolayer Bi Electrodeposition on Au(111): Surface X-ray Scattering and Current Transients. *J. Phys. Chem. B* **2004**, *108*, 1992–1998.
(7) Tao, N.-J.; Pan, J.; Li, Y.; Oden, P. I.; DeRose, J. A.; Lindsay, S. M. Initial Stage of Underpotential Deposition of Pb on Reconstructed and Unreconstructed Au(111). *Surf. Sci.* **1992**, *271*, 338–344.
(8) Chen, C.-h.; Washburn, N.; Gewirth, A. A. In Situ Atomic Force Microscope Study of Lead Underpotential Deposition on Au(111): Structural Properties of the Catalytically Active Phase. *J. Phys. Chem.* **1993**, *97*, 9754–9760.
(9) Chen, C.-h.; Gewirth, A. A. Correlation of Electrode Surface Structure with Activity toward Peroxide Electroreduction for Bismuth Monolayers on Gold(111). *J. Am. Chem. Soc.* **1992**, *114*, 5439–5440.
(10) Toney, M. F.; Gordon, J. G.; Samant, M. G.; Borges, G. L.; Melroy, O. R.; Yee, D.; Sorensen, L. B. In-Situ Atomic Structure of Underpotentially Deposited Monolayers of Pb and Tl on Au(111) and Ag(111): A Surface X-ray Scattering Study. *J. Phys. Chem.* **1995**, *99*, 4733–4744.
(11) Deakin, M. R.; Melroy, O. Underpotential Metal Deposition on Gold, Monitored in situ with a Quartz Microbalance. *J. Electroanal. Chem. Interf. Electrochem.* **1988**, *239*, 321–331.
(12) Ragoisha, G. A.; Bondarenko, A. S. Potentiodynamic Electrochemical Impedance Spectroscopy. *Electrochimica Acta* **2005**, *50*, 1553–1563.
(13) Stafford, G. R.; Bertocci, U. In Situ Stress and Nanogravimetric Measurements During Underpotential Deposition of Bismuth on (111)-Textured Au. *J. Phys. Chem. B* **2006**, *110*, 15493–15498.
(14) Melroy, O.; Kanazawa, K.; Gordon, J. G., II; Buttry, D. Direct Determination of the Mass of an Underpotentially Deposited Monolayer of Lead on Gold. *Langmuir* **1986**, *2*, 697–700.
(15) Crist, B. V. The International XPS Database. https://xpsdatabase.net/ (accessed 2025-04-09).
(16) He, B.; Tian, G.; Gou, J.; Liu, B.; Shen, K.; Tian, Q.; Yu, Z.; Song, F.; Xie, H.; Gao, Y.; Lu, Y.; Wu, K.; Chen, L.; Huang, H. Structural and Electronic Properties of Atomically Thin Bismuth on Au(111). *Surf. Sci.* **2019**, *679*, 147–153.
(17) Szczepanska, A.; Wan, G.; Cattelan, M.; Fox, N. A.; Vasiljevic, N. Surface Investigation on Electrochemically Deposited Lead on Gold. *Surfaces* **2019**, *2*, 56–68.
(18) Yeh, J. J.; Lindau, I. Atomic Subshell Photoionization Cross Sections and Asymmetry Parameters: $1 \leqslant Z \leqslant 103$. *At. Data Nucl. Data Tables* **1985**, *32*, 1–155.
(19) Ju, H.; Wang, J.; Liu, W.; Hao, J.; Li, M.; Xu, Y.; Wang, B.; He, S.; Mei, K.; Sue, A. C.-H.; Chen, K.; Jia, C.; Guo, X. Single-Molecule Characterization of van der Waals Contact between Alkane and Gold. *CCS Chem.* **2024**, *6*, 2704–2712.
(20) Haiss, W.; Martín, S.; Leary, E.; van Zalinge, H.; Higgins, S. J.; Bouffier, L.; Nichols, R. J. Impact of Junction Formation Method and Surface Roughness on Single Molecule Conductance. *J. Phys. Chem. C* **2009**, *113*, 5823–5833.

(21) Chen, H.; Li, Y.; Chang, S. Hybrid Molecular-Junction Mapping Technique for Simultaneous Measurements of Single-Molecule Electronic Conductance and Its Corresponding Binding Geometry in a Tunneling Junction. *Anal. Chem.* **2020**, *92*, 6423–6429.
(22) Chen, F.; Li, X.; Hihath, J.; Huang, Z.; Tao, N.-J. Effect of Anchoring Groups on Single-Molecule Conductance: Comparative Study of Thiol-, Amine-, and Carboxylic-Acid-Terminated Molecules. *J. Am. Chem. Soc.* **2006**, *128*, 15874–15881.
(23) Park, Y. S.; Whalley, A. C.; Kamenetska, M.; Steigerwald, M. L.; Hybertsen, M. S.; Nuckolls, C.; Venkataraman, L. Contact Chemistry and Single-Molecule Conductance: A Comparison of Phosphines, Methyl Sulfides, and Amines. *J. Am. Chem. Soc.* **2007**, *129*, 15768–15769.
(24) Huang, M.-J.; Hsu, L.-Y.; Fu, M.-D.; Chuang, S.-T.; Tien, F.-W.; Chen, C.-h. Conductance of Tailored Molecular Segments: A Rudimentary Assessment by Landauer formulation. *J. Am. Chem. Soc.* **2014**, *136*, 1832–1841.
(25) Gu, M.-W.; Lai, C.-T.; Ni, I-C.; Wu, C.-I; Chen, C.-h. Increased Surface Density of States at the Fermi Level for Electron Transport across Single-Molecule Junctions. *Angew. Chem. Int. Ed.* **2023**, *62*, e202214963.
(26) Li, C.; Pobelov, I.; Wandlowski, T.; Bagrets, A.; Arnold, A.; Evers, F. Charge Transport in Single Au | Alkanedithiol | Au Junctions: Coordination Geometries and Conformational Degrees of Freedom. *J. Am. Chem. Soc.* **2008**, *130*, 318–326.
(27) Ko, C.-H.; Huang, M.-J.; Fu, M.-D.; Chen, C.-h. Superior Contact for Single-Molecule Conductance: Electronic Coupling of Thiolate and Isothiocyanate on Pt, Pd, and Au. *J. Am. Chem. Soc.* **2010**, *132*, 756–764.
(28) Kim, C. M.; Bechhoefer, J. Conductive Probe AFM Study of Pt-Thiol and Au-Thiol Contacts in Metal-Molecule-Metal Systems. *J. Chem. Phys.* **2013**, *138*, 014707.
(29) Huber, R.; González, M. T.; Wu, S.; Langer, M.; Grunder, S.; Horhoiu, V.; Mayor, M.; Bryce, M. R.; Wang, C.; Jitchati, R.; Schönenberger, C.; Calame, M. Electrical Conductance of Conjugated Oligomers at the Single Molecule Level. *J. Am. Chem. Soc.* **2008**, *130*, 1080–1084.
(30) Hybertsen, M. S.; Venkataraman, L.; Klare, J. E.; Whalley, A. C.; Steigerwald, M. L.; Nuckolls, C. Amine-Linked Single-Molecule Circuits: Systematic Trends across Molecular Families. *J. Phys. Condens. Matter* **2008**, *20*, 374115.
(31) Chen, I-W. P.; Tseng, W.-H.; Gu, M.-W.; Su, L.-C.; Hsu, C.-H.; Chang, W.-H.; Chen, C.-h. Tactile-Feedback Stabilized Molecular Junctions for the Measurement of Molecular Conductance. *Angew. Chem. Int. Ed.* **2013**, *52*, 2449–2453.
(32) Liu, J.; Zhao, X.; Zheng, J.; Huang, X.; Tang, Y.; Wang, F.; Li, R.; Pi, J.; Huang, C.; Wang, L.; Yang, Y.; Shi, J.; Mao, B.-W.; Tian, Z.-Q.; Bryce, M. R.; Hong, W. Transition from Tunneling Leakage Current to Molecular Tunneling in Single-Molecule Junctions. *Chem* **2019**, *5*, 390-401.
(33) Fatemi, V.; Kamenetska, M.; Neaton, J. B.; Venkataraman, L. Environmental Control of Single-Molecule Junction Transport. *Nano Lett.* **2011**, *11*, 1988–1992.
(34) Kotiuga, M.; Darancet, P.; Arroyo, C. R.; Venkataraman, L.; Neaton, J. B. Adsorption-Induced Solvent-Based Electrostatic Gating of Charge Transport through Molecular Junctions. *Nano Lett.* **2015**, *15*, 4498–4503.
(35) Trouwborst, M. L.; Martin, C. A.; Smit, R. H.; Guedon, C. M.; Baart, T. A.; van der Molen, S. J.; van Ruitenbeek, J. M. Transition Voltage Spectroscopy and the Nature of Vacuum Tunneling. *Nano Lett.* **2011**, *11*, 614–617.
(36) van Veen, F. H.; Ornago, L.; van der Zant, H. S. J.; El Abbassi, M. Benchmark Study of Alkane Molecular Chains. *J. Phys. Chem. C* **2022**, *126*, 8801–8806.
(37) Landauer, R. Electrical Resistance of Disordered One-Dimensional Lattices. *Philos. Mag.* **1970**, *21*, 863–867.
(38) Meir, Y.; Wingreen, N. S. Landauer Formula for the Current through an Interacting Electron Region. *Phys. Rev. Lett.* **1992**, *68*, 2512–2515.
(39) Newns, D. M. Self-Consistent Model of Hydrogen Chemisorption. *Phys. Rev.* **1969**, *178*, 1123–1135.

(40) Cuevas, J. C.; Scheer, E. *Molecular Electronics: An Introduction to Theory and Experiment*; World Scientific: Singapore, 2010.
(41) Harrison, W. A. *Electronic Structure and the Properties of Solids: The Physics of the Chemical Bond*; Dover: New York, 1989.
(42) Slater, J. C.; Koster, G. F. Simplified LCAO Method for the Periodic Potential Problem. *Phys. Rev.* **1954**, *94*, 1498–1524.
(43) Grimley, T. B. The Indirect Interaction between Atoms or Molecules Adsorbed on Metals. *Proc. Phys. Soc.* **1967**, *90*, 751.
(44) Gu, M.-W.; Peng, H. H.; Chen, I-W. P.; Chen, C.-h. Tuning Surface *d* Bands with Bimetallic Electrodes to Facilitate Electron Transport across Molecular Junctions. *Nat. Mater.* **2021**, *20*, 658–664.
(45) Lin, G.-M.; Lin, C.-H.; Peng, H. H.; Hsiao, H.; Wang, T.-H.; Ho, C.-H.; Hsu, H.-F.; Chen, C.-h. Effect of the Chemical Potentials of Electrodes on Charge Transport across Molecular Junctions. *J. Phys. Chem. C* **2019**, *123*, 22009–22017.
(46) Vojvodic, A.; Nørskov, J. K.; Abild-Pedersen, F. Electronic Structure Effects in Transition Metal Surface Chemistry. *Top. Catal.* **2014**, *57*, 25–32.
(47) Hammer, B.; Nørskov, J. K. Theoretical Surface Science and Catalysis—Calculations and Concepts. *Adv. Catal.* **2000**, *45*, 71–129.
(48) Papaconstantopoulos, D. *Handbook of the Band Structure of Elemental Solids: From Z = 1 To Z = 112, Second Edition*; Springer New York, NY: 2015; pp 1–655.
(49) Liu, S.; Li, Z.; Wang, C.; Tao, W.; Huang, M.; Zuo, M.; Yang, Y.; Yang, K.; Zhang, L.; Chen, S.; Xu, P.; Chen, Q. Turning Main-Group Element Magnesium into a Highly Active Electrocatalyst for Oxygen Reduction Reaction. *Nat. Commun.* **2020**, *11*, 938.
(50) Liu, H.; Bai, Y.; Wu, M.; Yang, Y.; Wang, Y.; Li, L.; Hao, J.; Yan, W.; Shi, W. A Regenerable Bi-Based Catalyst for Efficient and Stable Electrochemical CO_2 Reduction to Formate at Industrial Current Densities. *Angew. Chem. Int. Ed.* **2024**, *63*, e202411575.
(51) Han, N.; Wang, Y.; Yang, H.; Deng, J.; Wu, J.; Li, Y.; Li, Y. Ultrathin Bismuth Nanosheets from in situ Topotactic Transformation for Selective Electrocatalytic CO_2 Reduction to Formate. *Nat. Commun.* **2018**, *9*, 1320.
(52) Froyen, S.; Harrison, W. A. Elementary Prediction of Linear Combination of Atomic Orbitals Matrix Elements. *Phys. Rev. B* **1979**, *20*, 2420–2422.
(53) Xin, H.; Schweitzer, N.; Nikolla, E.; Linic, S. Communications: Developing Relationships between the Local Chemical Reactivity of Alloy Catalysts and Physical Characteristics of Constituent Metal Elements. *J. Chem. Phys.* **2010**, *132*, 111101.
(54) Hammer, B.; Nørskov, J. K. Electronic Factors Determining the Reactivity of Metal Surfaces. *Surf. Sci.* **1995**, *343*, 211–220.
(55) Cao, X.; Tian, Y.; Ma, J.; Guo, W.; Cai, W.; Zhang, J. Strong *p-d* Orbital Hybridization on Bismuth Nanosheets for High Performing CO_2 Electroreduction. *Adv. Mater.* **2024**, *36*, 2309648.
(56) Xie, T.; Wang, P.; Tian, C.; Zhao, G.; Jia, J.; Zhao, C.; Wu, H. The Adsorption Behavior of Gas Molecules on Co/N Co–Doped Graphene. *Molecules* **2021**, *26*, 7700.
(57) Cao, A.; Wang, Z.; Li, H.; Elnabawy, A. O.; Nørskov, J. K. New Insights on CO and CO_2 Hydrogenation for Methanol Synthesis: The Key Role of Adsorbate-Adsorbate Interactions on Cu and the Highly Active MgO-Cu Interface. *J. Catal.* **2021**, *400*, 325–331.
(58) Lu, F.; Xie, W.; Yi, D.; Wang, Y.; Zhang, F.; Xu, Y.; Zhou, B.; Liu, S.; Wang, X.; Yao, J. Revealing the Role of *d* Orbitals of Transition-Metal-Doped Titanium Oxide on High-Efficient Oxygen Reduction. *CCS Chem.* **2021**, *3*, 180–188.
(59) Yang, Y.; Liu, J.; Wei, Z.; Wang, S.; Ma, J. Transition Metal-Dinitrogen Complex Embedded Graphene for Nitrogen Reduction Reaction. *ChemCatChem* **2019**, *11*, 2821–2827.
(60) Chen, H.; Wu, Q.; Wang, Y.; Zhao, Q.; Ai, X.; Shen, Y.; Zou, X. d–sp orbital Hybridization: A Strategy for Activity Improvement of Transition Metal Catalysts. *Chem. Comm.* **2022**, *58*, 7730–7740.

(61) Wu, Q.; Huang, B.; Dai, Y.; Heine, T.; Ma, Y. Main-Group Metal Elements as Promising Active Centers for Single-Atom Catalyst toward Nitric Oxide Reduction Reaction. *npj 2D Mater. Appl.* **2022**, *6*, 52.
(62) Obersteiner, V.; Egger, D. A.; Zojer, E. Impact of Anchoring Groups on Ballistic Transport: Single Molecule vs Monolayer Junctions. *J. Phys. Chem. C* **2015**, *119*, 21198–21208.
(63) Hong, W.; Manrique, D. Z.; Moreno-Garcia, P.; Gulcur, M.; Mishchenko, A.; Lambert, C. J.; Bryce, M. R.; Wandlowski, T. Single Molecular Conductance of Tolanes: Experimental and Theoretical Study on the Junction Evolution Dependent on the Anchoring Group. *J. Am. Chem. Soc.* **2012**, *134*, 2292–2304.
(64) Isshiki, Y.; Fujii, S.; Nishino, T.; Kiguchi, M. Fluctuation in Interface and Electronic Structure of Single-Molecule Junctions Investigated by Current Versus Bias Voltage Characteristics. *J. Am. Chem. Soc.* **2018**, *140*, 3760–3767.
(65) Brandbyge, M.; Mozos, J.-L.; Ordejón, P.; Taylor, J.; Stokbro, K. Density-Functional Method for Nonequilibrium Electron Transport. *Phys. Rev. B* **2002**, *65*, 165401.
(66) Smidstrup, S.; Stradi, D.; Wellendorff, J.; Khomyakov, P. A.; Vej-Hansen, U. G.; Lee, M.-E.; Ghosh, T.; Jónsson, E.; Jónsson, H.; Stokbro, K. First-Principles Green's-function Method for Surface Calculations: A Pseudopotential Localized Basis Set Approach. *Phys. Rev. B* **2017**, *96*, 195309.
(67) Tao, Y. T. Structural Comparison of Self-Assembled Monolayers of *n*-Alkanoic Acids on the Surfaces of Silver, Copper, and Aluminum. *J. Am. Chem. Soc.* **1993**, *115*, 4350–4358.
(68) Lin, S.-Y.; Chen, C.-h.; Chan, Y.-C.; Lin, C.-M.; Chen, H.-W. Self-Assembly of Alkanoic Acids on Gold Surfaces Modified by Underpotential Deposition. *J. Phys. Chem. B* **2001**, *105*, 4951–4955.
(69) He, P.; Daaoub, A. H. S.; Sangtarash, S.; Sadeghi, H.; Yoon, H. J. Thermopower in Underpotential Deposition-Based Molecular Junctions. *Nano Lett.* **2024**, *24*, 1988–1995.
(70) Liu, L.; Zhang, Q.; Tao, S.; Zhao, C.; Almutib, E.; Al-Galiby, Q.; Bailey, S. W. D.; Grace, I.; Lambert, C. J.; Du, J.; Yang, L. Charge Transport through Dicarboxylic-Acid-Terminated Alkanes Bound to Graphene–Gold Nanogap Electrodes. *Nanoscale* **2016**, *8*, 14507–14513.
(71) Huang, J.-R.; Huang, H.; Tao, C.-P.; Zheng, J.-F.; Yuan, Y.; Hong, Z.-W.; Shao, Y.; Niu, Z.-J.; Chen, J.-Z.; Zhou, X.-S. Controlling Contact Configuration of Carboxylic Acid-Based Molecular Junctions through Side Group. *Nanoscale Res. Lett.* **2019**, *14*, 253.
(72) Ansari, S. M.; Bhor, R. D.; Pai, K. R.; Sen, D.; Mazumder, S.; Ghosh, K.; Kolekar, Y. D.; Ramana, C. V. Cobalt Nanoparticles for Biomedical Applications: Facile Synthesis, Physiochemical Characterization, Cytotoxicity Behavior and Biocompatibility. *Appl. Surf. Sci.* **2017**, *414*, 171–187.
(73) Lin, S.-Y.; Tsai, T.-K.; Lin, C.-M.; Chen, C.-h.; Chan, Y.-C.; Chen, H.-W. Structures of Self-Assembled Monolayers of *n*-Alkanoic Acids on Gold Surfaces Modified by Underpotential Deposition of Silver and Copper: Odd−Even Effect. *Langmuir* **2002**, *18*, 5473–5478.
